# αVβ8 integrin targeting to prevent posterior capsular opacification

**DOI:** 10.1172/jci.insight.145715

**Published:** 2021-11-08

**Authors:** Mahbubul H. Shihan, Samuel G. Novo, Yan Wang, Dean Sheppard, Amha Atakilit, Thomas D. Arnold, Nicole M. Rossi, Adam P. Faranda, Melinda K. Duncan

**Affiliations:** 1 Department of Biological Sciences, University of Delaware, Newark, Delaware, USA.; 2 Lung Biology Center, Department of Medicine, and; 3 Department of Pediatrics, University of California, San Francisco, San Francisco, California, USA.

**Keywords:** Cell Biology, Ophthalmology, Drug therapy, Fibrosis

## Abstract

Fibrotic posterior capsular opacification (PCO), a major complication of cataract surgery, is driven by transforming growth factor–β (TGF-β). Previously, αV integrins were found to be critical for the onset of TGF-β–mediated PCO in vivo; however, the functional heterodimer was unknown. Here, β8 integrin–conditional knockout (β8ITG-cKO) lens epithelial cells (LCs) attenuated their fibrotic responses, while both β5 and β6 integrin–null LCs underwent fibrotic changes similar to WT at 5 days post cataract surgery (PCS). RNA-Seq revealed that β8ITG-cKO LCs attenuated their upregulation of integrins and their ligands, as well as known targets of TGF-β–induced signaling, at 24 hours PCS. Treatment of β8ITG-cKO eyes with active TGF-β1 at the time of surgery rescued the fibrotic response. Treatment of WT mice with an anti-αVβ8 integrin function blocking antibody at the time of surgery ameliorated both canonical TGF-β signaling and LC fibrotic response PCS, and treatment at 5 days PCS, after surgically induced fibrotic responses were established, largely reversed this fibrotic response. These data suggest that αVβ8 integrin is a major regulator of TGF-β activation by LCs PCS and that therapeutics targeting αVβ8 integrin could be effective for fibrotic PCO prevention and treatment.

## Graphical abstract

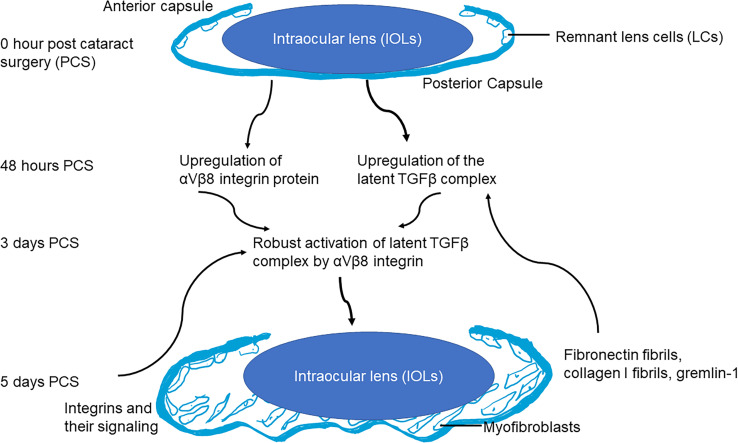

## Introduction

Cataracts, a major cause of blindness (1), are treated by surgical removal of opaque lens cells followed by implantation of an artificial intraocular lens (IOL) (1). However, months to years later, a significant proportion of patients experience an apparent recurrence of their cataract as posterior capsular opacification (PCO) (2, 3). PCO occurs when the remnant lens epithelial cells (LCs) left behind post cataract surgery (PCS) migrate into the optical axis and transition into a mixture of myofibroblasts and aberrant lens fiber cells (3). Approximately 25% of adult human and veterinary patients, and almost 100% of pediatric patients who do not receive prophylactic posterior capsulotomy, develop clinically significant PCO within months to years PCS (4). PCO in adults is treated by neodymium:YAG (Nd:YAG) laser capsulotomy (2), although this is often unsuitable/inconvenient for pediatric and veterinary therapy (4). As Nd:YAG laser capsulotomy can also cause side effects, including macular edema and retinal detachment, PCO prevention is desirable (2, 4, 5). Currently, the only US FDA–approved approach to prevent PCO utilizes prosthetic IOLs, which sequester remnant LCs to the capsular bag periphery, an innovation that delays, but often does not prevent, PCO (4).

Transforming growth factor–β (TGF-β) signaling mediates the epithelial-mesenchymal transition (EMT) of LCs to myofibroblasts (6). While TGF-β concentrations in the aqueous humor are high before surgery, most of this TGF-β is inactive (7). Using a mouse cataract surgery model, we previously demonstrated that canonical TGF-β signaling is not easily detected in LCs until 48 hours PCS, with robust activation initiating at 3 days PCS (8). However, the mechanisms by which cataract surgery results in TGF-β signaling activation are unknown.

Integrins, heterodimeric extracellular matrix (ECM) receptors consisting of 1 α and 1 β subunit, mediate cell/ECM attachment, cell migration, and force transmission (9). Integrins also crosstalk with growth factor signaling (10), including the TGF-β pathway (11, 12). Thus, integrins are potential therapeutic targets for PCO prevention and/or treatment (9). Previously, we found that αV integrins are critical for fibrotic PCO (13) consistent with their known roles in latent TGF-β activation (14, 15). Notably, the αV integrin subunit heterodimerizes with a variety of β integrins (16), 4 of which (β1, β5, β6, and β8) upregulate in LCs PCS with dynamics similar to αV (13). Since each αV integrin heterodimer binds different ligands, and is inhibited by different compounds (17), the identification of the β subunit that pairs with αV to drive PCO is critical to both the development of anti-PCO therapies and the investigation of operant signaling mechanisms. In this study, we demonstrate that the gene encoding the β8 integrin subunit is necessary for LCs to trigger TGF-β signaling and fibrotic responses PCS and show that an αVβ8 integrin function blocking antibody ameliorates PCS TGF-β signaling and fibrosis, suggesting a possible therapeutic approach for PCO prevention.

## Results

### Neither β_5_ nor β_6_ integrins are critical for LC fibrosis PCS; however, β_8_ integrin protein levels upregulate in a pattern correlating with TGF-β signaling induction in LCs PCS.

**Figure 1:**
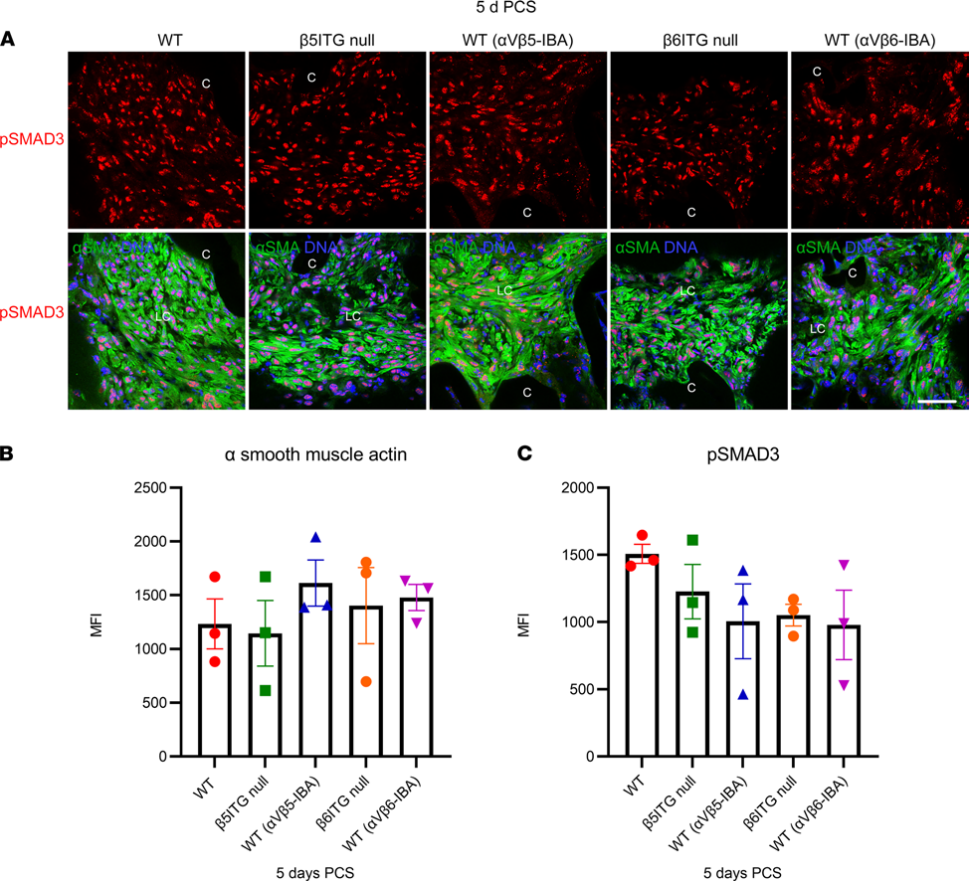
Neither β_5_ integrin nor β_6_ integrins are essential for lens cells to undergo fibrotic changes PCS. (**A**–**C**) Wild-type (WT), β_5_ integrin–null (β_5_ITG), and β_6_ integrin–null (β_6_ITG) mice were subjected to lens fiber cell removal, and α-SMA expression and TGF-β signaling (phosphorylated SMAD3, p-SMAD3) were measured 5 days later (5 d PCS). LCs from both mutant genotypes exhibited similar levels of SMAD3 phosphorylation and α-SMA induction compared with WT (α-SMA; β_5_ integrin–null, *P* = 0.999; β_6_ integrin–null, *P* = 0.988) (p-SMAD3; β_5_ integrin–null, *P* = 0.847; β_6_ integrin–null, *P* = 0.513). Similarly, administration of an α_V_β_5_ integrin function blocking antibody (α_V_β_5_-IBA) or α_V_β_6_ integrin function blocking antibody (α_V_β_6_-IBA) to WT mice did not affect these responses compared with WT (α-SMA; WT [α_V_β_5_-IBA], *P* = 0.830; WT [α_V_β_6_-IBA], *P* = 0.958); (p-SMAD3; WT [α_V_β_5_-IBA], *P* = 0.427; WT [α_V_β_6_-IBA], *P* = 0.382). Control mice were treated with an isotype-matched antibody (anti–human α_V_β_3_ integrin that does not cross-react with the mouse α_V_β_3_ integrin protein). Blue, DNA detected by Draq5; green, α-SMA; red, p-SMAD3; scale bar: 36 μm. Mean ± SEM is presented for 1 representative experiment (*n* = 3) of 2 independent experiments, with similar results; *P* values determined by 1-way ANOVA with Tukey’s post hoc test. Graph colors: (**B** and **C**) red (WT), green [β_5_ITG-null], blue (WT [α_V_β_5_-IBA]), orange (β_6_ITG-null), purple (WT [α_V_β_6_-IBA]). C, lens capsule; LC, lens cells; d, day; PCS, post cataract surgery; β_5_ITG, β_5_ integrin; β_6_ITG, β_6_ integrin.

Our prior study revealed that an α_V_ integrin is critical for the fibrotic response of LCs PCS (13). As α_V_ integrins are heterodimers between the α_V_ integrin subunit and one of several possible β integrins (16), we sought to identify which α_V_β integrin complex regulates the LC response to surgery. As β_5_ and β_6_ integrin levels upregulate in LCs PCS (13), and α_V_β_5_ and α_V_β_6_ integrins can regulate fibrosis (14, 18–20), we first characterized potential roles for β_5_ and β_6_ integrin in fibrotic PCO. β_5_ and β_6_ integrin–null adult lenses were transparent (not shown) and exhibited fibrotic responses, and canonical TGF-β signaling, at 5 days PCS similar to WT, as measured by the expression of a myofibroblast marker, α–smooth muscle actin (α-SMA; Figure 1, A and B; β_5_ integrin–null, *P* = 0.999; β_6_ integrin–null, *P* = 0.988), and SMAD2/3 phosphorylation (Figure 1, A and C; β_5_ integrin–null, *P* = 0.847; β_6_ integrin–null, *P* = 0.513). Similarly, systemic administration of either an α_V_β_5_-IBA or α_V_β_6_-IBA to WT mice following fiber cell removal did not alter the fibrotic response of LCs following lens injury (α-SMA; Figure 1, A and B; WT α_V_β_5_-IBA, *P* = 0.830; WT α_V_β_6_-IBA, *P* = 0.958) (TGF-β signaling PCS [p-SMAD2/3]; Figure 1, A and C; WT α_V_β_5_-IBA, *P* = 0.427; WT α_V_β_6_-IBA, *P* = 0.382). Overall, this suggests that neither α_V_β_5_ nor α_V_β_6_ integrins are essential for LC EMT PCS.

**Figure 2:**
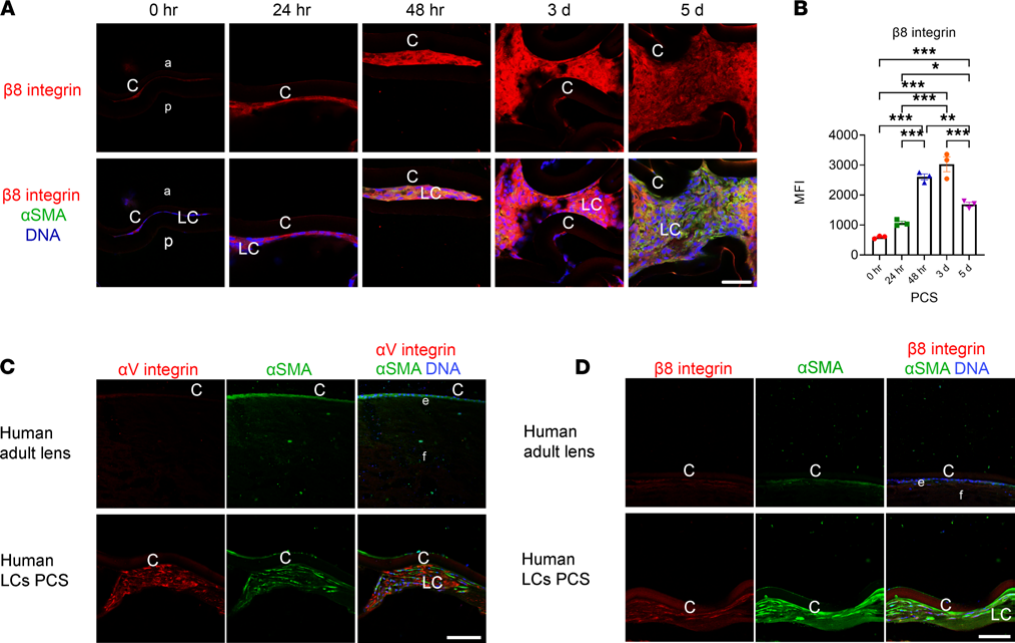
β_8_ integrin protein levels are upregulated in LCs PCS. (**A**) β_8_ integrin protein (red) levels are low in remnant LCs immediately PCS but upregulate in α-SMA–positive (green) remnant LCs and are robust by 3 days PCS. Scale bar (**A**): 36 μm. (**B**) Quantitation of the data from **A** with the mean fluorescence intensity (MFI) values measured in lens capsule–associated cells ± SEM is presented for 1 representative experiment of 2 independent experiments, with similar results; (*P ≤ 0.05; **P ≤ 0.01; ***P ≤ 0.001, *n* = 3); 1-way ANOVA with Tukey’s post hoc test. (**C** and **D**) Unoperated human LCs obtained from a cadaver eye exhibit modest α-SMA staining consistent with its previously reported presence in naive LCs (75), though they have little to no α_V_ integrin (red in **C**) or β_8_ integrin protein (red in **D**). In contrast, islands of LCs associated with a human lens capsule/IOL complex exhibit bright immunostaining of α_V_ integrin (**C**), β_8_ integrin (**D**), and the myofibroblast marker α-SMA (green). (Scale bars for **C** and **D**: 72 μm.) DNA (blue, **A**, **C**, and **D**). Graph colors: (**B**) red (WT 0 hours), green (WT 24 hours), blue (WT 48 hours), orange (WT 3 days), purple (WT 5 days). C, lens capsule; LC, remnant lens cells; a, anterior; p, posterior; e, epithelial cells; f, fiber cells; PCS, post cataract surgery; WT, wild-type; β_8_ITG-cKO, β_8_ integrin conditional knockout.

Next, the β_8_ integrin subunit was investigated as α_V_β_8_ integrin activates latent TGF-β in some circumstances (14, 15). At 0 hours PCS, remnant LCs expressed little β_8_ integrin protein (Figure 2, A and B), but its levels rose between 24 and 48 hours PCS (Figure 2, A and B; *P* ≤ 0.001); this was robust by 3 days PCS (Figure 2, A and B; *P* ≤ 0.001). β_8_ integrin expression was sustained through 5 days PCS (Figure 2, A and B; *P* ≤ 0.001) though its levels were significantly lower than 3 days PCS. In addition, both α_V_ and β_8_ integrin levels were similar between WT, β_5_ integrin–null, and β_6_ integrin–null capsular bags at 5 days PCS (Supplemental Figure 1; supplemental material available online with this article; https://doi.org/10.1172/jci.insight.145715DS1), suggesting that α_V_β_8_ integrin upregulation PCS is not dependent on either α_V_β_5_ integrin or α_V_β_6_ integrin. Further, neither α_V_ nor β_8_ integrin protein was detected in the intact human lens, but bright α_V_ integrin and β_8_ integrin staining was detected in LCs exhibiting high levels of α-SMA lining a human capsular bag collected several years after cataract surgery (Figure 2, C and D), indicating that α_V_β_8_ integrin upregulation in LCs PCS is conserved between humans and mice and may persist for extended times PCS.

**Figure 3:**
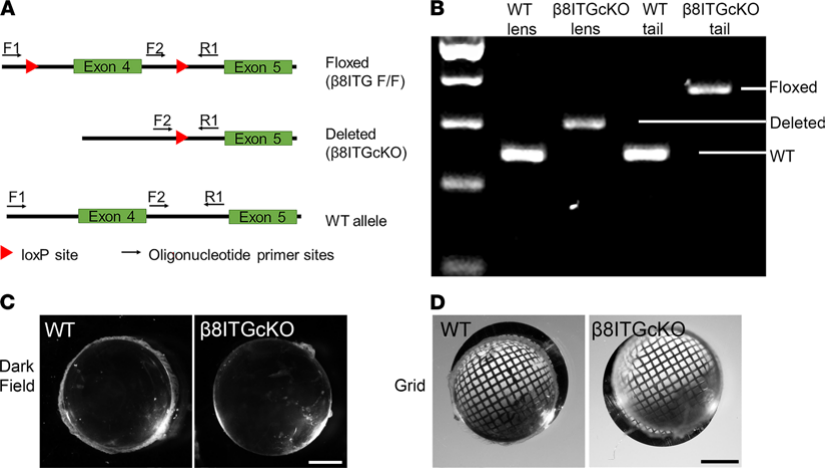
β_8_ integrin is not required for lens development. (**A**) Diagram of the β_8_ integrin gene showing the position of the *loxP* sites used to create β_8_ITG-cKO mice and the PCR primers used to assess the efficiency of deletion. (**B**) PCR results from DNA obtained from 8-week-old WT and β_8_ITG-cKO lenses and tails demonstrating successful deletion of the floxed gene fragment from β_8_ITG-cKO lenses. (**C** and **D**) Darkfield and grid analysis suggest that the adult lenses (12 months old) of β_8_ITG-cKO are transparent and have refractive properties similar to WT. (Scale bar for **C** and **D**: 17.5 mm.) The data are presented for 1 representative experiment (*n* = 3 each time) of 3 independent experiments, with similar results. WT, wild-type; β_8_ITG-cKO, β_8_ integrin conditional knockout.

Because β_8_ subunit protein level elevation observed in LCs PCS correlates with the timing of robust TGF-β signaling (8), we studied the function of α_V_β_8_ integrin in PCO by generating mice lacking the *Itgb8* gene from the lens (β_8_ITG-cKO) by mating mice carrying a floxed *Itgb8* allele (21) to mice harboring the lens-specific MLR10-Cre transgene (22) (Figure 3A). The deletion of the floxed region of *Itgb8* was confirmed by PCR analysis of genomic DNA isolated from adult lenses (Figure 3B) and by immunofluorescence of β_8_ integrin protein in WT and β_8_ITG-cKO LCs at 5 days PCS (Supplemental Figure 2). Adult β_8_ITG-cKO lenses (2–12 months old) are transparent (Figure 3C) and have refractive properties similar to WT (Figure 3D), suggesting that α_V_β_8_ integrin does not regulate adult lens structure. However, adult β_8_ITG-cKO lenses (8–14 months old) were 3.34% larger in diameter than WT lenses of similar age (*P* = 0.012), consistent with the phenotype of adult mouse lenses lacking the α_V_ integrin gene (*Itgav*) (13), suggesting that α_V_β_8_ integrin may regulate lens growth.

### Lenses lacking Itgb8 attenuate cell proliferation and fibrotic responses PCS while retaining their epithelial characteristics and ability to regenerate lens fiber cells.

**Figure 4:**
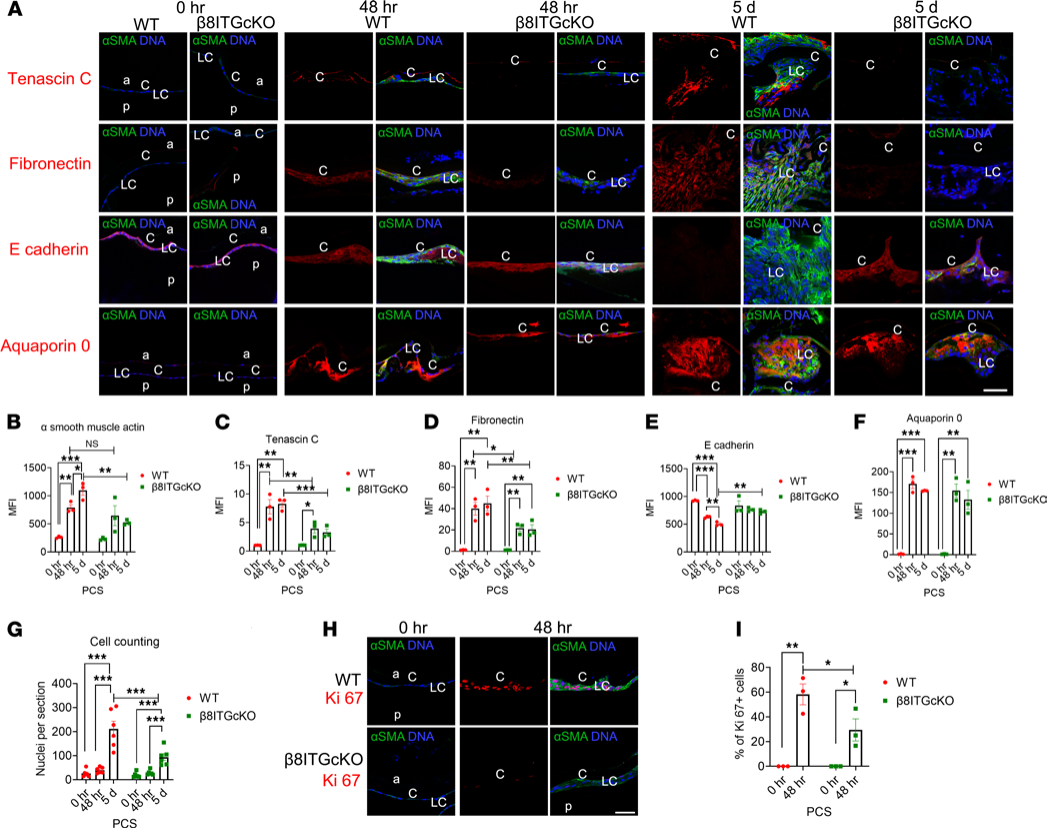
The response of LCs lacking the β_8_ integrin gene to lens fiber cell removal. (**A**–**F**) While the unpregulation of α-SMA in WT LCs persisted until 5 days from 48 hours PCS in WT LCs (*P* = 0.028), β8ITG-cKO LCs attenuated α-SMA (**A** and **B**; *P* = 0.001) upregulation at 5 days PCS and tenascin C (**A** and **C**; *P* = 0.005 [48 hours]; *P* ≤ 0.001 [5 days]) and fibronectin (**A** and **D**) upregulation at 48 hours (*P* = 0.022) and 5 days (*P* = 0.005) PCS. In contrast, E cadherin (**A** and **E**) significantly downregulated in WT LCs by 48 hours PCS, an effect sustained at 5 days PCS (*P* ≤ 0.001), but this did not occur in β_8_ITG-cKO LCs (*P* = 0.390). Fiber cell regeneration measured by aquaporin 0 expression (**A** and **F**) occurs to a similar extent in β_8_ITG-cKO and WT LCs PCS. (**G**) Counting of cell nuclei associated with lens capsular bags PCS reveals that fewer cells were associated with β_8_ITG-cKO capsular bags compared with WT 5 days PCS (*P* ≤ 0.001). (**H** and **I**) WT LCs induce expression of the cell cycle marker Ki67 by 48 hours PCS (*P* = 0.002) while a significantly lower proportion of β_8_ITG-cKO LCs are in the cell cycle at this time (*P* = 0.021). Scale bar: 35 μm. Tenascin C, fibronectin, E-cadherin, aquaporin 0, and Ki67 (red); α-SMA (green); DNA (blue). *n* = 3 except for **G**, which had *n* = 6. Values are expressed as mean ± SEM presented for 1 representative experiment of 3 independent experiments, with similar results; asterisks indicate statistically significant MFI/nuclei per section between WT and β_8_ITG-cKO at an indicated time point PCS or between 2 PCS time points (*P ≤ 0.05; **P ≤ 0.01; ***P ≤ 0.001); both Student’s 2-tailed *t* test (corrected for multiple comparisons using the Holm-Šídák method) and 1-way ANOVA with Tukey’s post hoc test (**B**–**G**) or Student’s 2-tailed *t* test (corrected for multiple comparisons using the Holm-Šídák method) (**I**). Graph colors: (**B**–**G** and **I**) red (WT), green (β_8_ITG-cKO). C, lens capsule; LC, lens cells; PCS, post cataract surgery; a, anterior; p, posterior.

The response of β_8_ITG-cKO LCs to lens fiber cell removal was tested by following the expression of the myofibroblast marker, α-SMA, and the fibrillar matrix proteins tenascin C and fibronectin (23) PCS (Figure 4, A–D). As expected, little to no α-SMA, tenascin C, or fibronectin protein were seen in or around remnant LCs associated with either WT or β_8_ITG-cKO capsular bags at 0 hours PCS. By 48 hours PCS, both WT and β_8_ITG-cKO LCs upregulated all 3 proteins; however, β_8_ITG-cKO LCs attenuated their upregulation of tenascin C (Figure 4, A and C; *P* = 0.005) and fibronectin (Figure 4, A and D; *P* = 0.022) protein compared with WT while α-SMA levels remained similar (Figure 4, A and B; *P* = 0.446). WT LCs further upregulated all 3 fibrotic proteins (Figure 4, A–D) (α-SMA, *P* ≤ 0.001; tenascin C, *P* = 0.002; fibronectin, *P* = 0.003) 5 days PCS. In contrast, β_8_ITG-cKO LCs still expressed less of these proteins 5 days PCS compared with WT (Figure 4, A and B–D) (α-SMA, *P* = 0.001; tenascin C, *P* ≤ 0.001; fibronectin, *P* = 0.005), suggesting *Itgb8* deletion from the lens inhibits the fibrotic response of remnant LCs PCS.

We next investigated the expression of an epithelial cell marker, E cadherin, to determine if β_8_ITG-cKO LCs retain their epithelial character PCS. As expected, both WT and β_8_ITG-cKO LCs expressed E cadherin protein at 0 hours PCS (Figure 4, A and E). However, by 48 hours PCS, E cadherin protein levels were downregulated in WT LCs (Figure 4, A and E; *P* ≤ 0.001), and this downregulation was sustained through 5 days PCS (Figure 4, A and E; *P* ≤ 0.001). In contrast, E cadherin levels were unchanged in β_8_ITG-cKO 48 hours (Figure 4, A and E; *P* = 0.651) and 5 days PCS (Figure 4, A and E; *P* = 0.390), suggesting that *Itgb8*-null LCs preserve their epithelial characteristics PCS.

Because the cells associated with β_8_ITG-cKO capsular bags at 5 days PCS are no longer in a monolayer along the capsule, the expression of aquaporin 0, a fiber cell preferred membrane protein, was investigated; some LCs differentiate into structurally aberrant lens fiber cells during PCO pathogenesis, contributing to “pearl-like” PCO when in the visual axis and Soemmering’s ring when restricted to the capsular bag periphery (3). Remnant LCs from WT and β_8_ITG-cKO mice expressed little aquaporin 0 (Figure 4, A and F) immediately PCS. By 48 hours PCS, WT and β_8_ITG-cKO capsular bags were associated with some aquaporin 0–expressing cells and robustly so by 5 days PCS (Figure 4, A and F; WT *P* ≤ 0.001; β_8_ITG-cKO *P* = 0.003), indicating that β_8_ITG-cKO LCs can differentiate into lens fiber-like cells PCS to a similar extent as WT.

Notably, fewer cells were associated with β_8_ITG-cKO capsular bags than WT at 5 days PCS (*P* ≤ 0.001) (Figure 4G). Because apoptosis was not detected in the capsular bags of either WT or β_8_ITG-cKO mice at any time PCS (data not shown), LC proliferation was investigated by following the expression of Ki67, a marker of all cell cycle stages except G_0_ (24). At 0 hours PCS, few to no remnant LCs were proliferating (Figure 4, H and I) in WT or β_8_ITG-cKO mice. In contrast, Ki67-positive LCs increased between 0 and 48 hours PCS in WT (Figure 4, H and I; *P* = 0.002) and β_8_ITG-cKO (Figure 4, H and I; *P* = 0.03). However, the WT capsular bag had a higher percentage of Ki67-positive cells compared with β_8_ITGcKOs at 48 hours PCS (Figure 4, H and I; *P* = 0.02), suggesting β_8_ITG-cKO LCs proliferate less PCS. These data suggest LCs lacking *Itgb8* attenuate their fibrotic response PCS while retaining their epithelial phenotype.

### RNA-Seq reveals genes associated with fibrosis and inflammation are differentially expressed in β_8_ITG-cKO LCs PCS.

Because phenotypic differences between WT and β_8_ITG-cKO LCs manifest by 48 hours PCS, we performed RNA-Seq on WT and β_8_ITG-cKO LCs at 24 hours PCS to gain insight into this phenotype’s proximal cause. This revealed 2312 genes differentially expressed in WT LCs at 24 hours PCS compared with 0 hours PCS (1273 genes upregulated, 1039 genes downregulated under criteria for biologically significant differences; ref. 25). As we previously reported (8, 26), the upregulated genes included those that participate in tissue inflammation (Supplemental Table 4) and fibrosis (Supplemental Table 5), while many genes important for lens structure and function were downregulated (Supplemental Table 6).

Comparison between WT and β_8_ITG-cKO LCs at 24 hours PCS revealed that 828 genes were differentially expressed under the biological significance criteria (25) (Supplemental Figure 3). Of these, 97 were upregulated in WT LCs by 24 hours PCS but not in β_8_ITG-cKO LCs (Supplemental Table 7). Consistent with β_8_ITG-cKO LCs’ muted fibrotic response PCS, several of these were associated with fibrotic disease while others regulate inflammation (Supplemental Table 8).

### β_8_ITG-cKO LCs fail to upregulate known TGF-β–responsive genes PCS, while attenuating their activation of TGF-β signaling, and this is rescued by adding active TGF-β1.

**Figure 5:**
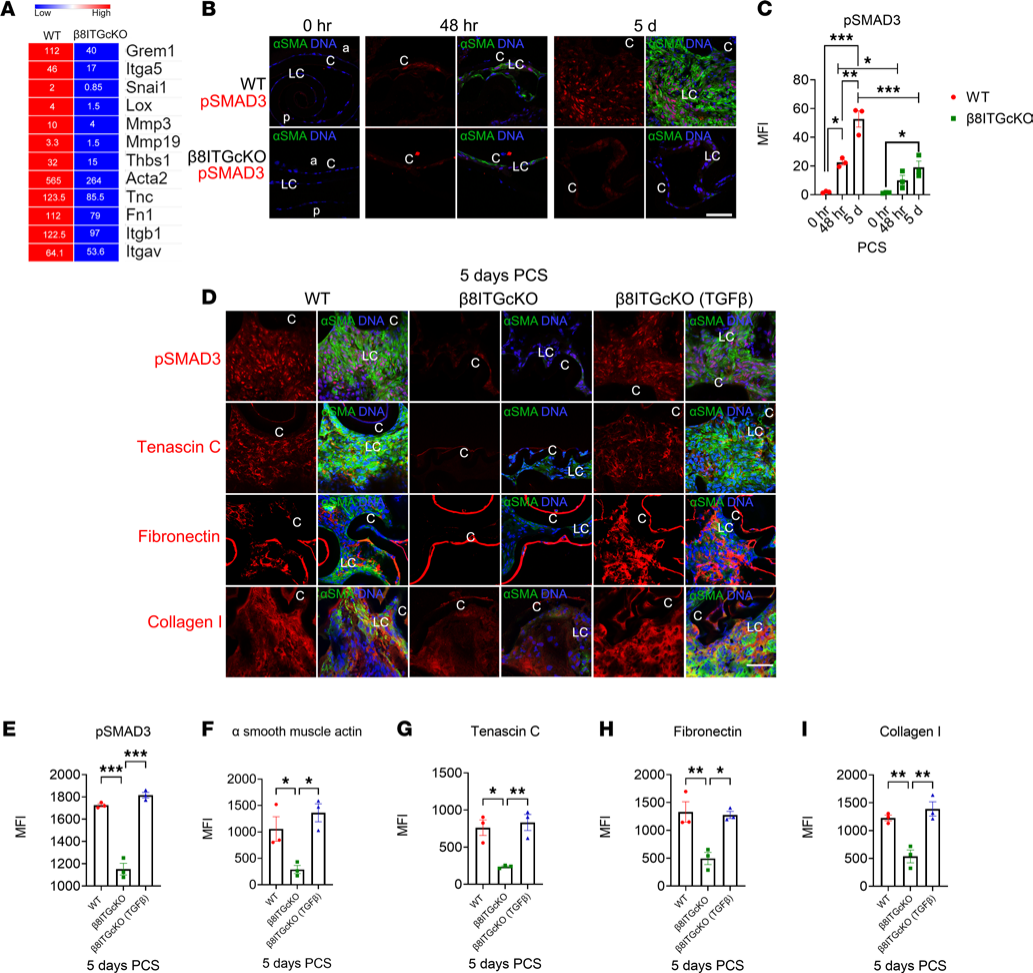
LCs lacking the β_8_ integrin gene fail to activate TGF-β signaling PCS, but this can be rescued by treatment with active TGF-β1. (**A**) Heatmap of genes known to participate in TGF-β pathways expressed at lower levels in β_8_ITG-cKO LCs at 24 hours PCS compared with WT. Expression levels are reported as fragments per kilobase million (FPKM). (**B** and **C**) SMAD3 phosphorylation (p-SMAD3) is detected in WT LCs by 48 hours PCS (*P* = 0.013), and this upregulates further by 5 days PCS (*P* ≤ 0.001; *P* ≤ 0.002). In contrast, β_8_ITG-cKO LCs exhibit attenuated SMAD3 phosphorylation 48 hours (*P* = 0.042) and 5 days PCS (*P* ≤ 0.001). (**D**–**I**) Treatment of β_8_ITG-cKO capsular bags with active TGF-β1 at the time of lens fiber cell removal rescued both p-SMAD3 levels (**D** and **E**; *P* ≤ 0.001), and the robust expression of α-SMA (**D** and **F**; *P* = 0.011), tenascin C (**D** and **G**; *P* *=* 0.007*)*, fibronectin (**D** and **H**; *P* = 0.012) and collagen I (**D** and **I**; *P* = 0.003) 5 days PCS. Scale bar: 35 μm. p-SMAD3, tenascin C, fibronectin, and collagen I (red); α-SMA (green); DNA detected by Draq5 (blue). All experiments had *n* = 3. Values are expressed as mean ± SEM presented for 1 representative experiment of 2 independent experiments, with similar results; asterisks indicate statistically significant MFI between WT and/or β_8_ITG-cKO and/or β_8_ITG-cKO (TGF-β) (*P ≤ 0.05; **P ≤ 0.01; ***P ≤ 0.001); both Student’s 2-tailed *t* test (corrected for multiple comparisons using the Holm-Šídák method) and 1-way ANOVA with Tukey’s post hoc test (**C**) or 1-way ANOVA with Tukey’s post hoc test (**E**–**I**). Graph colors: (**C**) red (different PCS time points of WT), green (different PCS time points of β_8_ITG-cKO); (**E**–**I**) red (WT), green (β_8_ITG-cKO), blue (β_8_ITG-cKO [TGF-β]. C, lens capsule; LC, remnant lens cells; PCS, post cataract surgery; a, anterior; p, posterior.

Notably, α_V_β_8_ integrin can activate latent TGF-β (27) while genes known either to regulate (gremlin-1 [*Grem1*], *Thbs1*, *Fn1,* *Itga5*) (23, 28–30), or to be regulated by TGF-β signaling (*Acta2*, *Tnc*) (23, 31), were differentially expressed in β_8_ITG-cKO LCs 24 hours PCS (Supplemental Table 8 and Figure 5A). Comparison with TGF-β–regulated genes in cultured cells (32) revealed 60 genes upregulated by TGF-β treatment exhibited attenuated upregulation in β_8_ITG-cKO LCs (Supplemental Table 9) but 47 genes downregulated by TGF-β treatment expressed at higher levels in β_8_ITG-cKO LCs at 24 hours PCS (Supplemental Table 10), suggesting α_V_β_8_ integrin affects TGF-β pathway activation PCS (OR 2.99, *P* = 6.3 × 10^–4^).

Thus, canonical TGF-β pathway activation PCS was determined in WT and β_8_ITG-cKO LCs by assaying the downstream effector of TGF-β signaling, p-SMAD2/3 (33) (Figure 5, B and C). As we previously reported (8), elevated p-SMAD2/3 levels were first detectable in WT LCs 48 hours PCS (Figure 5, B and C; *P* = 0.013) while this induction was attenuated in β_8_ITG-cKO LCs (Figure 5, B and C; *P* = 0.042). Further, while WT LCs further enhanced this signaling by 5 days PCS (Figure 5, B and C; *P* < 0.001), p-SMAD2/3 levels remained low in β_8_ITG-cKO LCs (Figure 5, B and C; *P* < 0.001), suggesting that TGF-β signaling PCS depends on the upregulation of β_8_ integrin expression.

Because active TGF-β induces LC conversion to myofibroblasts (6), and β_8_ITG-cKO LCs exhibit reduced TGF-β signaling PCS, we tested whether exogenous active TGF-β could rescue these defects (Figure 5, D–I). As active TGF-β1 treatment of β_8_ITG-cKO capsular bags caused robust induction of SMAD2/3 phosphorylation (Figure 5, D and E; *P* < 0.001) and α-SMA (Figure 5, D and F; *P* = 0.011), tenascin C (Figure 5, D and G; *P* = 0.007), fibronectin (Figure 5, D and H; *P* = 0.012), and collagen I (Figure 5, D and I; *P* = 0.003) 5 days PCS, α_V_β_8_ integrin may play a key role in activating TGF-β signaling in LCs PCS.

### Blocking the interaction of TGF-β latency associated peptide with α_V_β_8_ integrin in WT LCs phenocopies the attenuated fibrotic response and TGF-β signaling defects detected in β_8_ITG-cKO LCs PCS.

**Figure 6:**
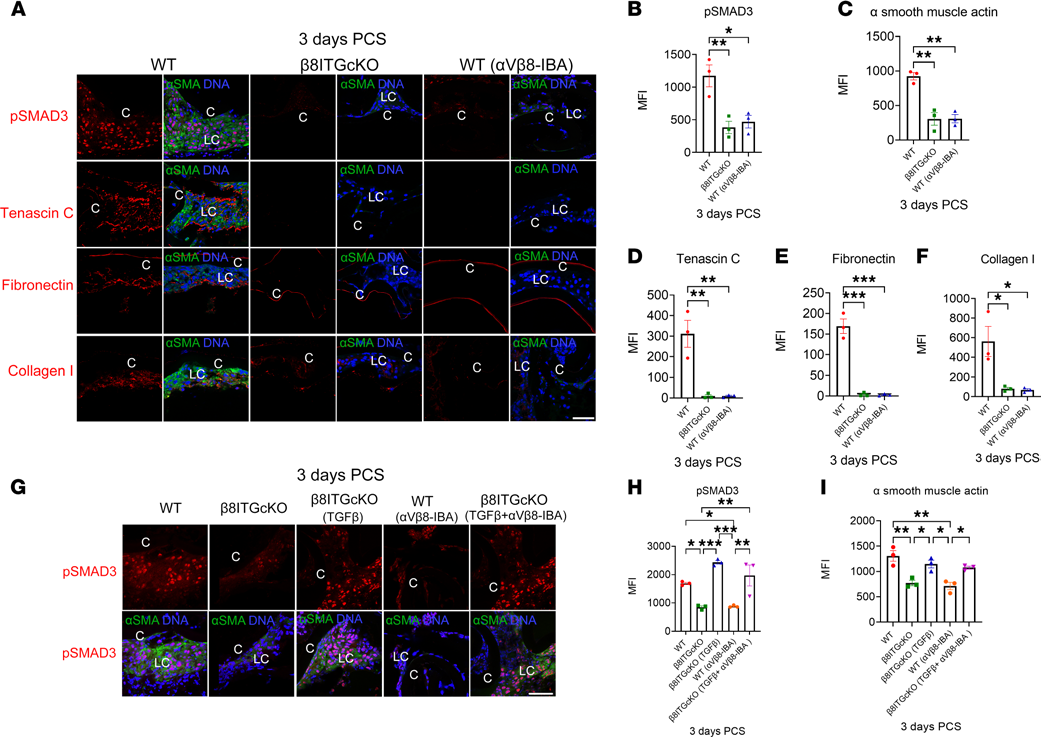
Treatment of WT mice with an α_V_β_8_-IBA prevents SMAD3 phosphorylation and fibrotic marker expression PCS. (**A**–**F**) The administration of an α_V_β_8_-IBA to WT mice inhibited TGF-β signaling and LC fibrotic responses at 3 days PCS to an extent similar to β_8_ITG-cKO as measured by its effect on SMAD3 phosphorylation (**A** and **B**; *P* = 0.017), and the expression levels of α-SMA (**A** and **C**; *P* = 0.002), tenascin C (**A** and **D**; *P* = 0.003), fibronectin (**A** and **E**; *P* ≤ 0.001), and collagen I (**A** and **F**; *P* = 0.019). (**G**–**I**) Coadministration of an α_V_β_8_-IBA with active TGF-β1 in β_8_ITG-cKO capsular bags at surgery rescues the attenuated fibrosis and TGF-β activation seen in β_8_ITG-cKO LCs (TGF-β+α_V_β_8_-IBA) as measured by α-SMA (**G** and **I**, *P* = 0.033) and p-SMAD3 (**G** and **H**, *P* = 0.008) at levels similar to treatment with active TGF-β1 alone (**G** and **I**, α-SMA, *P* = 0.951; **G** and **H**, p-SMAD3, *P* = 0.351). Scale bar: 35 μm. Controls were treated with an isotype-matched antibody (anti–human α_V_β_3_ integrin that does not cross-react with mouse α_V_β_3_ integrin); p-SMAD3, tenascin C, fibronectin, collagen I (red); α-SMA (green); DNA detected by Draq5/DAPI (blue). All experiments had *n* = 3. Values are expressed as mean ± SEM presented for 1 representative experiment of 2 independent experiments, with similar results; asterisks indicate statistically significant MFI between 2 groups at an indicated time point PCS (*P ≤ 0.05; **P ≤ 0.01; ***P ≤ 0.001); 1-way ANOVA with Tukey’s post hoc test. Graph colors: (**B**–**F**) red (WT), green (β_8_ITG-cKO), blue (WT [α_V_β_8_-IBA]); (**H** and **I**) red (WT), green (β_8_ITG-cKO), blue (β_8_ITG-cKO [TGF-β]), orange (WT [α_V_β_8_-IBA]), purple (β_8_ITG-cKO [TGF-β+ α_V_β_8_-IBA]). C, lens capsule; LC, remnant lens cells; PCS, post cataract surgery.

TGF-β is secreted from cells bound to its latency associated peptide (LAP) and latent binding proteins, forming the latent TGF-β complex (14). Upon secretion, the latent TGF-β complex is tethered to the ECM by binding to matrix proteins such as fibronectin (14, 23). The release of active TGF-β1 from the latent complex can be accomplished by the interaction of the LAP with integrins such as α_V_β_8_ (12, 14). Thus, we next tested whether α_V_β_8_ integrin function-blocking antibody (ADWA-11), which antagonizes LAP binding to α_V_β_8_ integrin (α_V_β_8_-IBA), thus blocking TGF-β activation (34), can influence the fibrotic response of LCs (Figure 6, A–F). Systemic treatment of WT mice at surgery with α_V_β_8_-IBA inhibited canonical TGF-β signaling measured by p-SMAD2/3 (Figure 6, A and B; *P* = 0.017) 3 days PCS, the time when robust TGF-β signaling is first detected in LCs (8). This correlated with attenuated fibrotic response of LCs 3 days PCS measured by α-SMA (Figure 6, A and C; *P* = 0.002), tenascin C (Figure 6, A and D; *P* = 0.003), fibronectin (Figure 6, A and E; *P* ≤ 0.001), and collagen I (Figure 6, A and F; *P* = 0.019) levels. As these attenuations were similar to that observed in β_8_ITG-cKO LCs (Figure 6, A–F), α_V_β_8_-IBA likely blocks TGF-β activation and subsequent fibrotic response of LCs PCS.

We confirmed that α_V_β_8_-IBA works at the level of TGF-β activation by coadministering active TGF-β1 (into the capsular bag) and α_V_β_8_-IBA (systemically) to β_8_ITG-cKO mice at surgery (Figure 6, G–I). As expected, active TGF-β1 rescued the TGF-β activation defect in β_8_ITG-cKO LCs and subsequent fibrosis at 3 days PCS even in the presence of α_V_β_8_-IBA measured by p-SMAD3 (Figure 6, G and H, P = 0.008) and α-SMA (Figure 6, G and I, P = 0.033) levels, and this phenocopied the rescue of fibrosis and TGF-β signaling in β_8_ITG-cKO capsular bags by active TGF-β1 treatment (Figure 6, G and H; p-SMAD3, *P* = 0.351; Figure 6, G and I; α-SMA, *P* = 0.951). This confirms that α_V_β_8_-IBA reduces LC fibrosis PCS by blocking the interaction of TGF-β LAP with α_V_β_8_ integrin.

**Figure 7:**
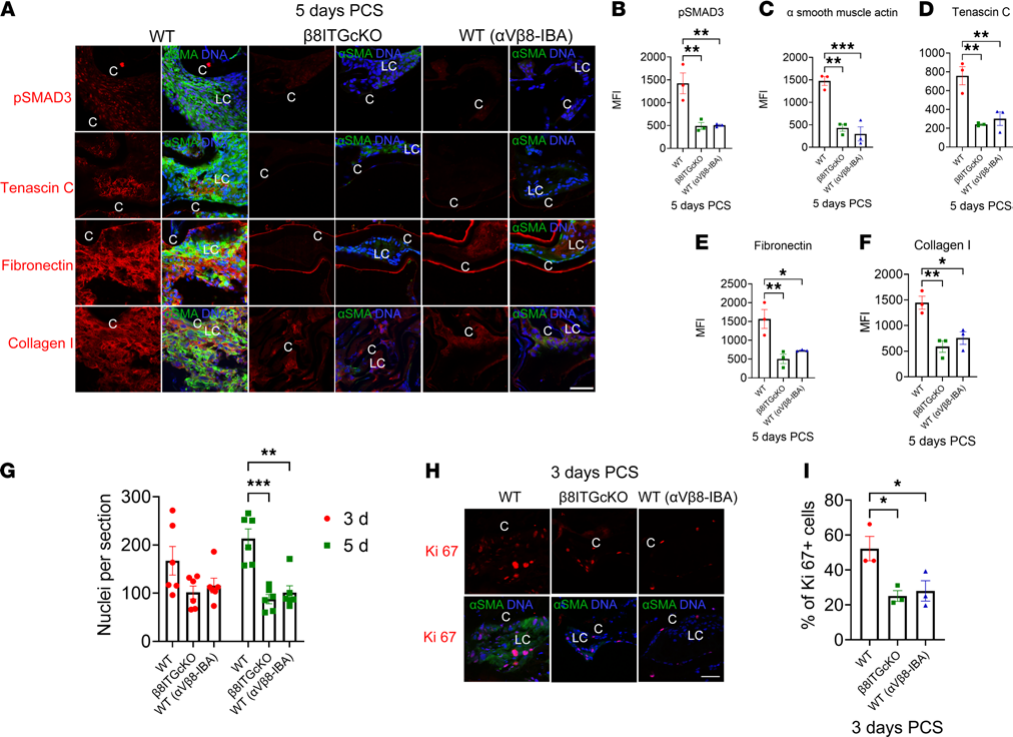
Treatment of WT mice with an α_V_β_8_-IBA causes sustained reduction of fibrosis and proliferation PCS. (**A**–**F**) A single systemic treatment of WT mice with α_V_β_8_-IBA causes sustained reduction of fibrosis through 5 days PCS as measured by the low levels of SMAD3 phosphorylation (**A** and **B**; *P* = 0.008) and α-SMA (**A** and **C**; *P* ≤ 0.001), tenascin C (**A** and **D**; *P* = 0.008), fibronectin (**A** and **E**; *P* = 0.025), and collagen I (**A** and **F**; *P* = 0.016) protein production. (**G**) Fewer cells were associated with β_8_ITG-cKO (*P* ≤ 0.001) and WT (α_V_β_8_-IBA) (*P* = 0.002) capsular bags compared with WT control at 5 days PCS. (**H** and **I**) WT LCs induce expression of the cell cycle marker Ki67 at 3 days PCS while a significantly lower proportion of β_8_ITG-cKO (*P* = 0. 031) and WT (α_V_β_8_-IBA) LCs (*P* = 0.048) are in the cell cycle at this time point. Scale bar: 35 μm. Control mice were treated with an isotype-matched antibody (anti–human α_V_β_3_ integrin that does not cross-react with the mouse α_V_β_3_ integrin protein); p-SMAD3, tenascin C, fibronectin, collagen I, and Ki67 (red); α-SMA (green); DNA detected by Draq5/DAPI (blue). All experiments had *n* = 3 except for **G**, which had *n* = 6. Values are expressed as mean ± SEM presented for 1 representative experiment of 2 independent experiments, with similar results; asterisks indicate statistically significant MFI/nuclei per section between 2 groups at an indicated time point PCS (*P ≤ 0.05; **P ≤ 0.01; ***P ≤ 0.001); 1-way/2-way ANOVA with Tukey’s post hoc test. Graph colors: (**B**–**F**) red (WT), green (β_8_ITG-cKO), blue (WT [α_V_β_8_-IBA]); (**G**) red — 3 days PCS for 3 conditions (WT [β_8_ITG-cKO]) (WT [α_V_β_8_-IBA]), green — 5 days PCS for 3 conditions (WT, [β_8_ITG-cKO]) (WT [α_V_β_8_-IBA]); (**I**) red (WT), green (β_8_ITG-cKO), blue (WT [α_V_β_8_-IBA]). C, lens capsule; LC, remnant lens cells; PCS, post cataract surgery; α_V_β_8_-IBA, α_V_β_8_ integrin function blocking antibody.

Next, we tested if a single treatment with α_V_β_8_-IBA at the time of fiber removal reduces LC fibrosis later PCS (Figure 7, A–F). Thus, α_V_β_8_-IBA was administered to WT mice at cataract surgery, and the samples were harvested 5 days PCS (when LCs exhibit sustained fibrotic responses and robust TGF-β signaling; ref. 23). α_V_β_8_-IBA–treated WT mice exhibited attenuated TGF-β signaling and fibrotic responses measured by reduced p-SMAD3 (Figure 7, A and B; *P* = 0.008) and α-SMA (Figure 7, A and C; *P* ≤ 0.001), tenascin C (Figure 7, A and D; *P* = 0.008), fibronectin (Figure 7, A and E; *P* = 0.025), and collagen I (Figure 7, A and F; *P* = 0.016) levels until at least 5 days PCS. Further, like β_8_ITG-cKO capsular bags, α_V_β_8_-IBA–treated WT capsular bags exhibited less LC proliferation 3 days PCS (Figure 7, G–I; β_8_ITG-cKO, *P* = 0.031; α_V_β_8_-IBA *P* = 0.048) than controls. This supports the idea that blockade of TGF-β activation at the time of lens injury is sufficient to prevent the fibrotic transformation of LCs.

**Figure 8:**
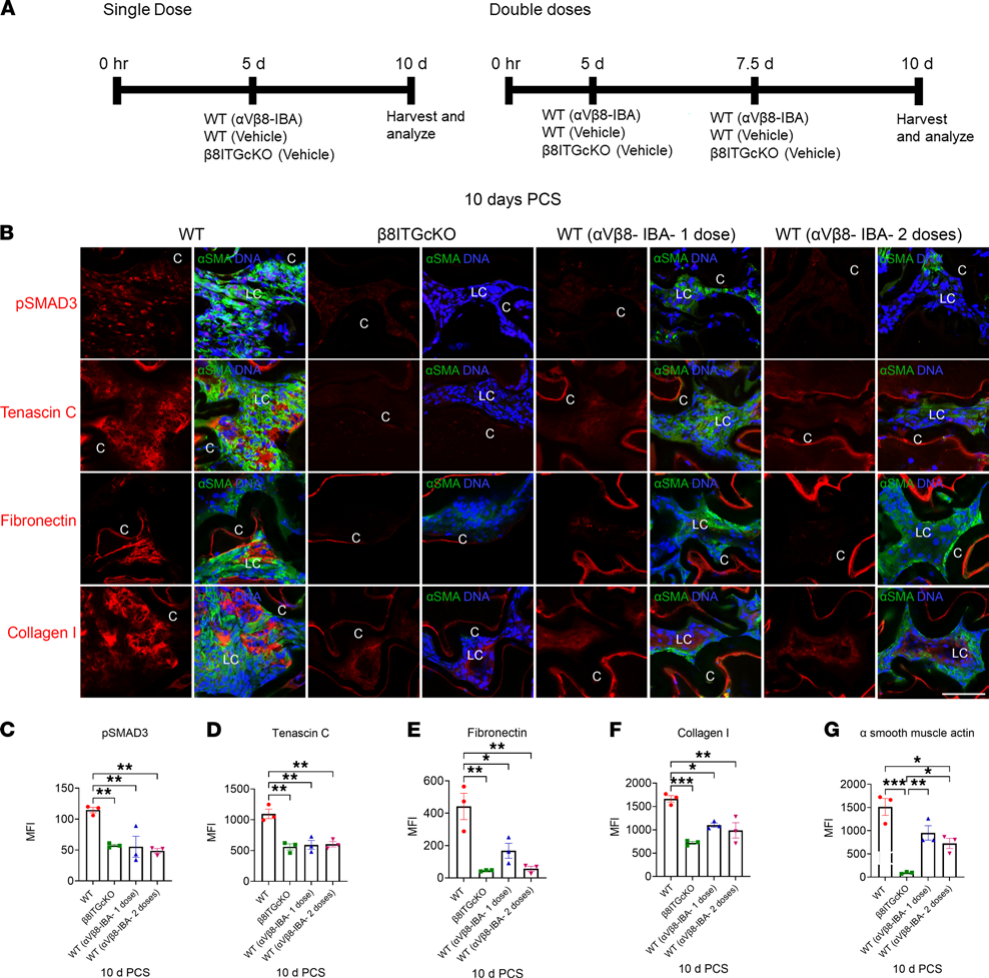
An α_V_β_8_-IBA can halt or reverse LC fibrosis PCS. (**A**) Dosing regimen for α_V_β_8_-IBA administration. (**B** and **C**) Treatment with α_V_β_8_-IBA starting at 5 days PCS inhibited SMAD3 phosphorylation (**B** and **C**; WT vs. WT α_V_β_8_-IBA-1 dose *P* = 0.007) (**B** and **C**; WT vs. WT α_V_β_8_-IBA-2 doses *P* = 0.004), at 10 days to levels similar to β_8_ITG-cKO LCs (**B** and **C**; WT vs. β_8_ITG-cKO *P* = 0.009; β_8_ITG-cKO vs. WT α_V_β_8_-IBA-1 dose *P* = 0.999; β_8_ITG-cKO vs. WT α_V_β_8_-IBA-2 doses *P* = 0.903). Tenascin C (**B** and **D**; WT vs. WT α_V_β_8_-IBA-1 dose *P* = 0.002; WT vs. WT α_V_β_8_-IBA-2 doses *P* = 0.002), fibronectin (**B** and **E**; WT vs. WT α_V_β_8_-IBA-1 dose *P* = 0.015; WT vs. WT α_V_β_8_-IBA-2 doses *P* = 0.002) and collagen I (**B** and **F**; WT vs. WT α_V_β_8_-IBA-1 dose *P* = 0.012; WT vs. WT α_V_β_8_-IBA-2 doses *P* = 0.004) staining was reduced at 10 days PCS to levels similar to β_8_ITG-cKO. While α_V_β_8_-IBA-1 dose did not decrease α-SMA levels at 10 days PCS, 2 doses of α_V_β_8_-IBA were effective (**B** and **G**; WT vs. WT α_V_β_8_-IBA-2 doses *P* = 0.013). Scale bar: 35 μm. Controls were treated with an isotype-matched antibody (anti–human α_V_β_3_ integrin that does not cross-react with mouse α_V_β_3_ integrin). p-SMAD3, tenascin C, fibronectin, and collagen I (red); α-SMA (green); DNA (blue). All experiments had *n* = 3. Values are expressed as mean ± SEM presented for 1 representative experiment of 2 independent experiments, with similar results; asterisks indicate statistically significant MFI between WT and/or β_8_ITG-cKO and/or WT (α_V_β_8_-IBA-1 dose) and/or WT (α_V_β_8_-IBA-2 doses) (*P ≤ 0.05; **P ≤ 0.01; ***P ≤ 0.001); 1-way ANOVA with Tukey’s post hoc test. Graph colors: (**C**–**G**) red (WT), green (β_8_ITG-cKO), blue (WT α_V_β_8_-IBA- 1 dose), purple (WT α_V_β_8_-IBA- 2 doses). C, lens capsule; LC, lens cells; PCS, post cataract surgery, α_V_β_8_-IBA, α_V_β_8_ integrin blocking antibody.

Finally, we investigated whether α_V_β_8_-IBA could arrest or reverse fibrotic PCO once established. Lens fibers were removed from WT mice, and 5 days PCS, they were treated systemically with either a single dose of α_V_β_8_-IBA, or 2 doses of α_V_β_8_-IBA given at 5 and 7.5 days PCS. Samples were harvested 10 days PCS (Figure 8A). Single-dose α_V_β_8_-IBA given 5 days PCS was sufficient to significantly attenuate SMAD2/3 phosphorylation 10 days PCS (Figure 8, B and C; WT vs. WT α_V_β_8_-IBA-1 dose *P* = 0.007) to a similar extent as β_8_ITG-cKO LCs (Figure 8, B and C; WT vs. β_8_ITG-cKO *P* = 0.009). Similarly, fibrotic marker protein levels were also significantly attenuated in α_V_β_8_-IBA–treated mice 10 days PCS compared with vehicle, and these attenuations were similar to those observed in β_8_ITG-cKO LCs (Figure 8, B and D; tenascin C WT vs. WT α_V_β_8_-IBA-1 dose *P* = 0.002; WT vs. β_8_ITG-cKO *P* = 0.001) (Figure 8, B and E; fibronectin WT vs. WT α_V_β_8_-IBA-1 dose *P* = 0.015; WT vs. β_8_ITG-cKO *P* = 0.002) (Figure 8, B and F; collagen I WT vs. WT α_V_β_8_-IBA-1 dose *P* = 0.012; WT vs. β_8_ITG-cKO *P* ≤ 0.001).

However, single-dose α_V_β_8_-IBA given 5 days PCS was insufficient to significantly attenuate α-SMA protein levels (Figure 8, B and G; WT vs. WT α_V_β_8_-IBA-1 dose *P* = 0.067). Notably, 2 doses of α_V_β_8_-IBA given 5 and 7.5 days PCS did significantly decrease α-SMA protein levels 10 days PCS (Figure 8, B and G; WT vs. WT α_V_β_8_-IBA-2 doses *P* = 0.013). However, α-SMA protein levels were still higher in both treatment groups (Figure 8, B and G; WT α_V_β_8_-IBA -1 dose *P* = 0.008) and (Figure 8, B and G; WT α_V_β_8_-IBA -2 doses, *P* = 0.038) LCs compared with β_8_ITG-cKO LCs 10 days PCS. This suggests α_V_β_8_-IBA therapy after LC fibrosis was established halted canonical TGF-β signaling, which substantially reduced fibrosis even in the presence of some degree of EMT.

Because 3 experimental approaches revealed that TGF-β activation by α_V_β_8_ integrin is a core mechanism of PCO development, next we characterized the regulatory relationship between α_V_β_8_ integrin–mediated TGF-β activation and 2 other PCO regulators, gremlin-1 and ECM binding integrins.

### Induction of α_V_β_8_ integrin is required for LCs to upregulate gremlin-1 levels PCS, though gremlin-1 does not rescue induction of canonical TGF-β signaling and fibrotic gene expression in β_8_ITG-cKO LCs.

Previously, we reported that gremlin-1, best known as a BMP antagonist (35), upregulates in LCs by 48 hours PCS and rescues the defects in sustained canonical TGF-β signaling observed in LCs lacking the fibronectin gene (23). Here, RNA-Seq revealed that *Grem1* mRNA levels were upregulated 170-fold in WT LCs at 24 hours PCS yet attenuated 3-fold in β_8_ITG-cKO LCs (Supplemental Tables 5 and 8 and Figure 4A). Consistent with these data, gremlin-1 protein levels were sharply upregulated in LCs associated with WT capsular bags by 3 days PCS (Supplemental Figure 4, A and B; *P* ≤ 0.001) while this upregulation was attenuated in β_8_ITG-cKO (Supplemental Figure 4, A and B; *P* = 0.002) and WT (α_V_β_8_-IBA) (Supplemental Figure 4, A and B; *P* ≤ 0.001) LCs. This trend was also observed 5 days PCS as gremlin-1 protein levels were significantly lower in β_8_ITG-cKO (Supplemental Figure 4, C and D; *P* = 0.017) and WT (α_V_β_8_-IBA) (Supplemental Figure 4, C and D; *P* = 0.022) LCs compared with WT, while the addition of active TGF-β1 to β_8_ITG-cKO eyes rescued gremlin-1 protein expression compared with β_8_ITG-cKO (vehicle) (Supplemental Figure 4, C and D; *P* = 0.004), suggesting that α_V_β_8_ integrin expression by LCs is critical for gremlin-1 upregulation PCS.

Gremlin-1 is an agonist of the canonical TGF-β pathway (36, 37) and rescues the defect in canonical TGF-β signaling observed in PCS LCs lacking the fibronectin gene (23). However, treating β_8_ITG-cKO mice with recombinant gremlin-1 at cataract surgery did not rescue LC fibrosis (Supplemental Figure 5, A–F). This suggests that gremlin-1–induced LC fibrosis may require the autocrine activation of TGF-β signaling, consistent with studies in other cell types (38, 39).

### Upregulation of integrin expression and signaling by LCs depends on α_V_β_8_ integrin–mediated TGF-β signaling.

**Figure 9:**
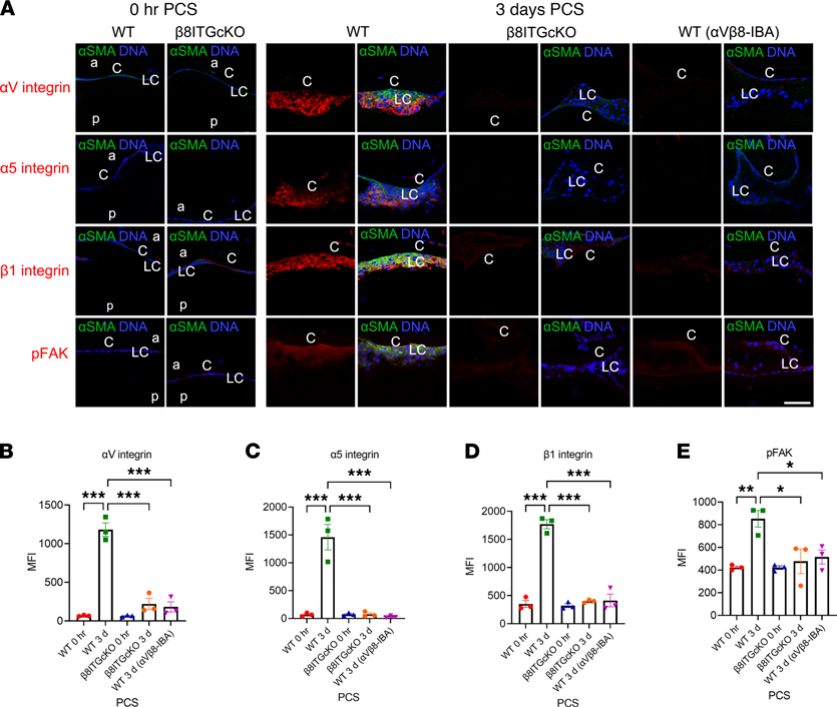
The dynamics of integrin expression and integrin signaling relative to canonical TGF-β signaling 3 days PCS. (**A**–**E**) WT LCs upregulate α_V_ integrin, α_5_ integrin, and β_1_ integrin as well as phosphorylated focal adhesion kinase (p-FAK) levels at 3 days PCS while β_8_ITG-cKO LCs fail to do so (**A** and **B**, α_V_ integrin, *P* < 0.001; **A** and **C**, α_5_ integrin, *P* < 0.001; **A** and **D**, β_1_ integrin, *P* < 0.001; **A** and **E**, p-FAK, *P* = 0.015). Like β_8_ITG-cKO, WT LCs treated with α_V_β_8_-IBA show attenuated expression of integrins and p-FAK levels compared with WT LCs (**A** and **B**, α_V_ integrin, *P* < 0.001; **A** and **C**, α_5_ integrin, *P* < 0.001; **A** and **D**, β_1_ integrin, *P* < 0.001; **A** and **E**, p-FAK, *P* = 0.029). Scale bar: 35 μm. Control mice were treated with an isotype-matched antibody (anti–human α_V_β_3_ integrin that does not cross-react with the mouse α_V_β_3_ integrin protein). α_V_ integrin, α_5_ integrin, β_1_ integrin, and p-FAK (red); α-SMA (green); DNA detected by Draq5/DAPI (blue). All experiments had *n* = 3. Values are expressed as mean ± SEM presented for 1 representative experiment of 2 independent experiments, with similar results; asterisks indicate statistically significant MFI between WT and/or β_8_ITG-cKO and/or WT (α_V_β_8_-IBA) at an indicated time point PCS or between 2 PCS time points. (*P ≤ 0.05; **P ≤ 0.01; ***P ≤ 0.001); 1-way ANOVA with Tukey’s post hoc test. Graph colors: (**B**–**E**) red (WT 0 hours), green (WT 3 days), blue (β_8_ITG-cKO 0 hours), orange (β_8_ITG-cKO 3 days), purple (WT 3 days α_V_β_8_-IBA). C, lens capsule; LC, remnant lens cells; a, anterior; p, posterior; PCS, post cataract surgery; α_V_β_8_-IBA, α_V_β_8_ integrin blocking antibody.

**Figure 10:**
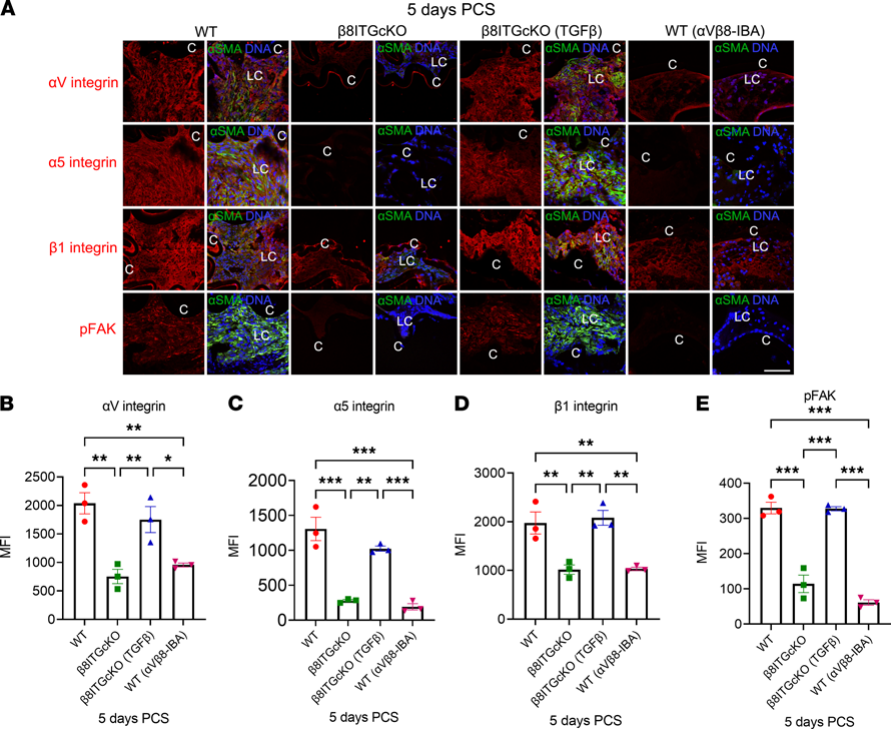
Upregulation of integrin expression and signaling 5 days PCS by LCs depends on α_V_β_8_ integrin. (**A**–**E**) Compared with WT LCs, both β_8_ITG-cKO (**A** and **B**, α_V_ integrin, *P* = 0.002; **A** and **C**, α_5_ integrin, *P* < 0.001; **A** and **D**, β_1_ integrin, *P* = 0.007; **A** and **E**, p-FAK, *P* < 0.001) and WT LCs treated with α_V_β_8_-IBA (**A** and **B**, α_V_ integrin, *P* = 0.006; **A** and **C**, α_5_ integrin, *P* < 0.001; **A** and **D**, β_1_ integrin, *P* = 0.008; **A** and **E**, p-FAK, *P* < 0.001) show attenuated levels for all 3 integrins and p-FAK at 5 days PCS. The addition of active TGF-β1 to β_8_ITG-cKO capsular bags rescues the attenuated integrin and p-FAK levels detected in β_8_ITG-cKO capsular bags (**A** and **B**, α_V_ integrin, *P* = 0.010; **A** and **C**, α_5_ integrin, *P* = 0.002; **A** and **D**, β_1_ integrin, *P* = 0.004; **A** and **E**, p-FAK, *P* < 0.001). Scale bar: 35 μm. Control mice were treated with an isotype-matched antibody (anti–human α_V_β_3_ integrin that does not cross-react with the mouse α_V_β_3_ integrin); α_V_ integrin, α_5_ integrin, β_1_ integrin and p-FAK (red), α-SMA (green), DNA detected by Draq5/DAPI (blue). All experiments had *n* = 3. Values are expressed as mean ± SEM presented for 1 representative experiment of 2 independent experiments, with similar results; asterisks indicate statistically significant MFI between WT and/or β_8_ITG-cKO and/or β_8_ITG-cKO (TGF-β) and/or WT (α_V_β_8_-IBA) at an indicated time point PCS or between 2 PCS time points. (*P ≤ 0.05; **P ≤ 0.01; ***P ≤ 0.001); 1-way ANOVA with Tukey’s post hoc test. Graph colors: (**B**–**E**) red (WT), green (β_8_ITG-cKO), blue (β_8_ITG-cKO [TGF-β]), purple (WT [α_V_β_8_-IBA]). C, lens capsule; LC, remnant lens cells; PCS, post cataract surgery; α_V_β_8_-IBA, α_V_β_8_ integrin blocking antibody.

Crosstalk between integrins and TGF-β signaling is well documented (12). Notably, LCs elevate the protein levels of α_5_β_1_ integrin and several α_V_ integrins in response to lens injury or TGF-β treatment (Figure 9) (9, 13, 23). Either deletion of the β_8_ integrin gene from LCs or treatment of capsular bags with a function blocking antibody against α_V_β_8_ integrin prevents the upregulation of α_5_, β_1_, and α_V_ integrin expression and increased p-FAK, a readout of integrin signaling PCS, at 3 and 5 days PCS (Figures 9 and 10). The addition of active TGF-β1 to β_8_ITG-cKO capsular bags rescued the attenuated integrin expression and p-FAK signaling detected in β_8_ITG-cKO LCs (Figure 10) 5 days PCS compared with vehicle-treated β_8_ITG-cKO LCs. Our findings indicate that α_V_β_8_ integrin is essential for upregulation of TGF-β signaling in LCs PCS, which drives subsequent upregulation of integrin expression and FAK signaling.

## Discussion

Fibrosis-mediated organ damage and failure are among the major causes of natural death worldwide because no effective therapies prevent or treat fibrosis (40). While TGF-β signaling often drives tissue fibrosis (6, 14), this pathway is difficult to target due to its complex regulation and diverse roles in normal biology (12). Integrins regulate the TGF-β pathway via their roles in latent TGF-β activation, and ability to mediate TGF-β effects, as integrin expression is often regulated by TGF-β signaling (12, 15, 41). Thus, integrins are promising therapeutic targets for organ fibrosis, and several integrin blocking agents are undergoing clinical trials (17, 42). The α_V_ integrins are particularly promising targets for antifibrotic therapies because blocking this class of integrins can ameliorate fibrosis in several organs (43, 44).

Previously, we reported that α_V_ integrin gene deletion from the lens prevents EMT of LCs, potentially due to their inability to initiate TGF-β signaling PCS (13). However, the identity of the β integrin subunit participating with α_V_ integrin was not known as multiple β subunits capable of heterodimerizing with the α_V_ integrin subunit are upregulated by LCs PCS (13). We could not study the role of α_V_β_1_ integrin in the LC response to injury in this study despite its known roles in wound healing (45), as we previously found that the *Itgb1* gene is essential for lens development and homeostasis, leading adult mice lacking β_1_ integrin expression in the lens to be severely microphthalmic/anophthalmic (46–48). However, the deletion of neither β_5_, β_6_, nor β_8_ integrin from the lens resulted in obvious lens defects, which made it possible to characterize their role in regulating the LC response to cataract surgery in vivo.

As previously reported, mice homozygous for β_5_ or β_6_ integrin deletions are viable (49, 50), while this investigation found that LCs from these mice underwent normal fibrotic responses PCS, indicating that neither α_V_β_5_ nor α_V_β_6_ integrins are critical for fibrotic PCO. While these results were initially surprising as these integrins can participate in latent TGF-β activation (18, 51), α_V_β_5_ and α_V_β_6_ integrins’ roles in fibrotic disease are tissue and insult specific (43, 49, 52). Thus, we investigated α_V_β_8_ integrin as (a) its expression rapidly upregulates in mouse LCs PCS and it is found on fibrotic human LCs at extended times PCS; (b) it can regulate tissue fibrosis and inflammation via binding to the RGD sequence present in the LAP of TGF-β1 and TGF-β3 (53), which activates these latent complexes via either formation of a ternary complex with membrane-type matrix metalloproteinase 1 (27), which is upregulated in LCs PCS, or traction-mediated activation (14); and (c) TGF-β1 and TGF-β3 mRNA levels are both upregulated in LCs PCS (8, 23).

### Integrin α_V_β_8_ adhesion to the LAP of latent TGF-β complexes.

TGF-β activation by α_V_β_8_ integrin plays roles in development, fibrosis, inflammation, and wound closure (14, 15). Notably, *itgav* deletion from the lens impedes TGF-β signaling and fibrotic responses PCS (13), and here we show that deletion of *itgb8* from the lens (β_8_ITG-cKO) phenocopies this result. As the addition of active TGF-β1 rescued the attenuated TGF-β signaling and fibrotic responses observed in β_8_ITG-cKO capsular bags PCS, this suggests that α_V_β_8_ integrin–mediated activation of latent TGF-β is critical for the development of fibrotic PCO. This hypothesis is supported by the observation that treatment of WT mice with ADWA-11, which specifically inhibits the adhesion of LAP to α_V_β_8_ integrin (34), potently inhibits the activation of TGF-β signaling and subsequent fibrotic responses of LCs to lens fiber cell removal.

### α_V_β_8_ integrin regulates both the EMT and LC proliferation in response to lens injury.

This study explored 3 potential mechanisms by which the *Itgb8* gene deletion from the lens and blocking of α_V_β_8_ integrin interaction with LAP inhibit the upregulation of proteins expressed by myofibroblasts and TGF-β signaling PCS. As LC apoptosis was apparently absent in both WT or β_8_ITG-cKO LCs PCS, the very large decrease in LC proliferation at 2 days and 3 days, and cell number at 5 days, PCS in β_8_ITG-cKO and WT (α_V_β_8_-IBA) in concert with the reduction of fibrotic marker mRNAs in β_8_ITG-cKO LCs at 24 hours PCS, and reductions in fibrotic marker protein expression later PCS, suggest that the α_V_β_8_ integrin/TGF-β signaling axis regulates both EMT and proliferation of LCs PCS, leading to fibrotic PCO.

### α_V_β_8_ integrin crosstalk with other integrins, and their signaling, in PCO.

Feedforward mechanisms between α_V_ integrins and TGF-β signaling have been previously described (12). Upon activation by α_V_ integrins, the TGF-β homodimer binds to the type II TGF-β receptor to initiate Smad2/3 phosphorylation, leading to increased expression of α_V_ integrins and other fibrotic markers. These newly formed integrins can liberate more TGF-β from latent complexes, sustaining and reinforcing TGF-β–induced fibrosis (12). Indeed, LCs lacking α_V_β_8_ integrin attenuate the upregulation of α_V_, α_5_, and β_1_ integrin expression and FAK phosphorylation PCS while treatment with active TGF-β1 reverses these defects. These findings have 2 implications: 1) targeting α_V_β_8_ integrin could suffice to prevent TGF-β activation in PCO; 2) the resulting attenuation of fibronectin fibril deposition (likely mediated by α_5_β_1_ integrin) may contribute to the long-term prevention of fibrotic PCO, as we have previously reported that fibronectin assembly is required for sustained LC fibrosis PCS (23).

### α_V_β_8_ integrin as a player in the gene regulatory network driving PCO.

Lens injury/cataract surgery results in the EMT of LCs to myofibroblasts that proliferate, migrate, contract the lens capsule, and produce a fibrotic matrix, which all contribute to the degradation of patient vision PCS (3). Most studies of this process start by considering the role of active TGF-β in this phenotypic conversion, though less attention has been paid to how cataract surgery initiates this process (8). Here we report that upregulation of α_V_β_8_ integrin levels on LCs PCS is an important step in their reprogramming into myofibroblasts and suggest that this reprogramming starts 1–2 days before upregulation of detectable canonical TGF-β signaling in these cells, as the upregulation of the mRNAs encoding numerous profibrotic proteins was attenuated in β_8_ITG-cKO LCs 24 hours PCS. As many of these genes have been previously reported to be TGF-β responsive in other cell types (Supplemental Tables 9 and 10), this observation supports our prior model, which proposed that a small upregulation in the protein levels of a TGF-β–activating integrin could lead to a feedforward loop that can rapidly induce autocrine TGF-β–mediated fibrosis (12).

Notably, the profibrotic modulators whose upregulation is attenuated in β_8_ITG-cKO 24 hours PCS included gremlin-1, a BMP signaling antagonist (35), and TGF-β signaling agonist (23, 28, 36), which usually upregulates sharply by 24 hours PCS. We previously found that LCs lacking the fibronectin gene exhibited greatly attenuated gremlin-1, p-SMAD2/3, and fibrotic marker upregulation PCS, while treatment of capsular bags with exogenous gremlin-1 rescued the ability of fibronectin-null LCs to undergo fibrotic responses (23). However, while both gremlin-1 expression and p-SMAD2/3 signaling are attenuated in β_8_ITG-cKO LCs, treatment of these cells with exogenous gremlin-1 did not rescue either p-SMAD2/3 signaling or the fibrotic response of β_8_ITG-cKO LCs, though exogenous treatment of these cells with active TGF-β1 rescued the fibrotic response. This suggests that gremlin-1 is not eliciting its response directly via the TGF-β receptor and instead may facilitate latent TGF-β activation by an as-yet-unknown mechanism. Alternatively, there could be differences in the requirements for gremlin-1 at different times PCS, with α_V_β_8_ integrin’s ability to activate TGF-β signaling at early times PCS kick-starting gremlin-1 expression so that it can influence TGF-β signaling later PCS (23). This concept is supported by work on other cell types suggesting that early activation of endogenous TGF-β is critical for gremlin-1 to exert its profibrotic response later in fibrotic disease (38, 39). Further study is required to elucidate the molecular mechanisms and therapeutic potential (if any) of gremlin-1 in PCO.

### α_V_β_8_ integrin blockade in halting the onset and progression of fibrotic PCO.

The emerging role of α_V_β_8_ integrin in TGF-β1 (and likely TGF-β3) activation has led to intense research into both antibody and small molecule inhibitors of α_V_β_8_ integrin–TGF-β interactions for the treatment (and monitoring) of fibrotic and neoplastic disease (15, 16, 34, 54). In most proof-of-principle experiments for this approach, these drugs prevent the onset of disease in animals, though this would not be clinically efficacious in most cases as fibrotic damage is often extensive by the time clinical symptoms manifest (40). However, treatment before the onset of fibrotic disease could be a viable option for anti-PCO therapy as the initiating insult (cataract surgery) is known, and the site of fibrosis is accessible during surgery, making local administration of the drug feasible. Here we validate this approach in an animal model of PCO as we found that the treatment of mice at the time of surgery with an anti–α_V_β_8_ integrin function blocking antibody prevented the development of the fibrotic sequelae of lens fiber cell removal and thus presumably fibrotic PCO. Our preclinical study supports the idea that this blocking antibody may be useful to halt fibrotic progression in patients who have already developed PCO. However, it should be mentioned that although the levels of p-SMAD3 activation and other fibrotic protein levels reversed in LCs to levels similar to the unoperated lens when mice were treated after fibrosis was established, α-SMA protein levels were still elevated, which may result from its relative stability as the half-life of α-SMA protein in cells is 72 hours (55).

### Limitations of this study for clinical translation as a PCO preventative.

While data presented here suggest that therapeutics blocking α_V_β_8_ integrin’s ability to activate TGF-β have great promise in preventing fibrotic PCO, unanswered questions remain. First, the function blocking antibody therapy used in this study was administered to the animals intravenously as the systemic administration of ADWA-11 showed no evidence of toxicity in prior studies (34, 56, 57), and a humanized version of ADWA-11 did not elicit notable systemic toxicity in mice and Cynomolgus monkeys treated for over 1 month at doses more than 5 times that used here (56, 57). However, attempts to directly inject the ADWA-11 into the mouse capsular bag at surgery did not block the fibrotic transformation of LCs. This may result from the rapid turnover of aqueous humor and the limited amount of antibody we were able to administer into the anterior chamber, leading the local concentration of antibody to quickly drop below the therapeutic dose (58, 59). In the future, we envision that function blocking drugs against α_V_β_8_ integrin could be administered locally in the eye at surgery either as a slow-release suspension (60) added to a dropless cataract surgery preparation (61) or by coating or soaking the IOL (62), which would reduce drug costs and opportunity for systemic side effects. Second, the invasive nature of the mouse “cataract surgery” model used in this study makes it difficult to assess drug effects on other ocular structures. Additional studies in rabbit and other animal models of cataract surgery that are more similar to what is performed in humans are necessary to assess ocular toxicity explicitly. Finally, while α_V_β_8_ integrin blockade appeared to reverse fibrotic ECM deposition associated with mouse capsular bags at 5 days PCS, we expect this matrix to still be relatively immature at this time and thus relatively susceptible to turnover. Future work will be needed to determine whether anti–α_v_β_8_ integrin therapeutics can reverse fibrosis in more established fibrotic conditions where the scar tissue has developed abundant amounts of cross-linked collagen.

### Summary.

This study established that α_V_β_8_ integrin is essential for LCs to transition to myofibroblasts following lens injury, likely through its ability to activate latent TGF-β1 and/or TGF-β3. Blocking α_V_β_8_ integrin binding to ligands via antibody blockade phenocopied the response of β_8_ITG-cKO LCs to lens fiber cell removal, establishing α_V_β_8_ integrin as a potentially novel therapeutic target to prevent PCO. PCO is a prevalent complication of cataract surgery (1, 3), especially in children. While there are no FDA-approved pharmacological agents available to prevent PCO, this preclinical study suggests inhibition of α_V_β_8_ integrin is a promising approach to PCO prevention. Further, the reversal of LC fibrosis by α_V_β_8_ integrin blockade suggests that therapeutics targeting α_V_β_8_ integrin have the potential to not just arrest the progression of fibrotic disease but also even reverse it.

## Methods

### Animals.

All mice were maintained under pathogen-free conditions at the University of Delaware animal facility under a 14-hour light/10-hour dark cycle. Animals of both sexes were used in these experiments, and no sex-dependent effects were noted, consistent with our prior report (63).

Mice homozygous for a null mutation in the β_5_ integrin gene were originally obtained from The Jackson Laboratory (*Itgb5^tm1Des^*, mixed [C57BL/6J 129/Sv], from the University of California, San Francisco; UCSF) (49). Homozygous β_6_ integrin–null mice (*Itgb6^tm1Des^*) on a 129Svems genetic background were obtained from Xiaozhu Huang (UCSF) (50). Mice lacking β_8_ integrin subunit gene from the lens (β_8_ITG-cKO) were created by mating mice harboring an integrin β_8_ allele on a mixed C57BL/6J 129/Sv background in which exon 4 is flanked by *loxP* sites (*Itgb8^tm2Lfr^*; obtained from UCSF) (21) with MLR10-cre mice on a C57BL/6<har> genetic background, which express Cre recombinase in all LCs from the lens vesicle stage onward (22) (obtained from Michael Robinson, Miami University, Oxford, Ohio, USA), backcrossed to C57BL/6<har> for over 10 generations.

### Human eyes.

Transparent lenses (30 ± 2 years of age) were obtained from Lions Eye Bank of Oregon (Portland, Oregon, USA), and aphakic donor eyes were obtained from the Minnesota Lions Eye Bank (Minneapolis, Minnesota, USA) as part of their cadaver eye tissue procurement programs. Intact lenses or lens capsular bag/IOL implant complexes were isolated, fresh frozen in OCT medium, and prepared for immunofluorescence experiments as described below.

### Genotyping and PCR.

DNA was isolated from tail snips or whole lenses using the PureGene Tissue and Mouse Tail kit (Gentra Systems) as described (13) and genotyped by PCR using primers described in Supplemental Table 1 (64, 65). The deletion of exon 4 of the integrin β_8_ gene from the lens was confirmed by PCR analysis of genomic DNA isolated from adult lenses using the primers described in Supplemental Table 1 (65).

### Morphological analysis.

Lens clarity was determined by viewing isolated lenses using darkfield optics while lens optical properties were assessed by placing lenses on a 200-mesh electron microscopy grid as described previously (66).

### Mouse cataract surgery model.

Surgical removal of lens fiber cells to mimic human cataract surgery was performed in adult mice as previously described (13, 67). Briefly, adult mice were anesthetized, a central corneal incision was made, and the entire lens fiber cell mass removed by a sharp forceps, leaving behind an intact lens capsule. Mice were sacrificed for analysis at time intervals ranging from 24 hours to 10 days PCS (13).

### RNA-Seq and bioinformatics.

Samples from WT (C57BL/6) and β_8_ITG-cKO lenses subjected to cataract surgery (3 biological replicates for each condition, 5 capsules per replicate) were harvested at 0 hours and 24 hours PCS and frozen on dry ice, and RNA was harvested using RNeasy Mini Kit (50) from QIAGEN (catalog 74104) (23). RNA libraries were prepared using the SMARTer Stranded Total RNA-Seq Kit-Pico Input Mammalian (Takara Bio USA, Inc.) and sequenced by DNA Link, USA, on a NovaSeq 6000 (Illumina). Read pairs corresponding to RNA fragments were enumerated as FPKM by Cuffdiff. Biologically significant differentially expressed genes (DEGs) were defined as those exhibiting statistically significant changes (FDR ≤ 0.05), a change in mRNA level greater than 2 FPKM between conditions, fold change greater than 2 in either the positive or negative direction, and expression levels in either condition that were 2 FPKM or greater (8, 23, 25). Heatmaps were generated using the Morpheus tool (68). RNA-Seq data were submitted to the National Center for Biotechnology Information’s Gene Expression Omnibus under accession number GSE145492.

A list of 1603 TGF-β–responsive genes in cultured cells (32) was filtered to consider only those statistically significant (FDR ≤ 0.05) genes with consistent directions of change between treatments, leaving 1390 genes as likely to be directly TGF-β responsive. This filtered list was then compared with the list of genes differentially expressed in WT LCs between 0 and 24 hours PCS and between WT and β_8_ITG-cKO LCs 24 hours PCS (FDR ≤ 0.05) to discover what proportion of the DEG list had the potential to be influenced by TGF-β signaling the first day PCS.

### Phenotypic rescue by active TGF-β1 and gremlin-1.

Rescue experiments were performed by instilling active recombinant human TGF-β1 protein (5 μL of 0.1 ng/μL TGF-β1 in balanced saline solution; BSS; R&D Systems; catalog 240-B) or recombinant human gremlin-1 protein (5 μL of 1 ng/μL gremlin-1 in BSS; R&D Systems, catalog 5190-GR) or the BSS vehicle alone into the lens capsular bags of β_8_ITG-cKO mice immediately following removal of the lens fibers as previously described (23).

### Tail vein injection of α_V_β_5_, α_V_β_6_, and α_V_β_8_ integrin function blocking antibodies.

Tail vein injection of α_V_β_5_, α_V_β_6_, and α_V_β_8_ integrin function blocking antibodies was performed as described (69). Briefly, a single dose of ALULA (α_V_β_5_ integrin blocking) (70), 3G9 (α_V_β_6_ integrin blocking) (71), or ADWA-11 (α_V_β_8_ integrin blocking) (34) was administered to WT mice at 20 mg/kg in PBS via lateral tail vein injection immediately following removal of the lens fiber cells from 1 eye. In another experiment, either a single dose of ADWA-11 (at 5 days PCS) or 2 doses of ADWA-11 (at 5 days PCS and 7.5 days PCS) was administered to WT mice. Control animals were treated with an isotype-matched antibody (anti–human α_V_β_3_ integrin that does not cross-react with the mouse α_V_β_3_ integrin protein) at 20 mg/kg in PBS. All the integrin function blocking antibodies were obtained from UCSF.

### Immunofluorescence.

The details of sample preparation and immunofluorescence were described previously (23, 72). Supplemental Table 2 describes the primary antibodies, blocking buffer compositions, incubation times, and dilutions used in this study, and Supplemental Table 3 lists the secondary antibodies and DNA dyes used in this study. Each experiment/time point was replicated using at least 3 biologically independent specimens (3–5 mice, at least 2 sections per mouse). Fluorescently labeled slides were visualized using Zeiss LSM780 or Zeiss LSM880 confocal microscopes (Carl Zeiss Inc.), and comparisons of images were made between slides imaged using identical imaging parameters (73). In some cases, the brightness and contrast were adjusted to allow viewing on diverse computer screens; however, these adjustments were made identically for all images within a particular time course. Negative controls were prepared and imaged to exclude nonspecific staining by the secondary antibodies or channel bleed-through as previously described (23, 72, 73).

### ImageJ (NIH) quantification and statistics.

Immunofluorescence images were quantified by determining the MFI of lens capsule–associated tissue viewed in 3 randomly chosen confocal images from biologically independent samples using ImageJ (v1.52P, NIH) (73). The average number of lens capsule–associated nuclei/section was analyzed by ImageJ using 6 randomly chosen immunofluorescence images from each PCS time point from at least 3 biologically independent samples as described (73, 74).

The diameter of adult lenses was determined by dissecting both lenses from 3 WT and 3 β_8_ITG-cKO mice and photographing them in brightfield using a Zeiss STEMI SV 11 dissecting microscope. The diameter of each lens was measured in 2 perpendicular axes using ImageJ, then averaged for statistical analysis.

All statistics were assessed using either 2-tailed Student’s *t* test (corrected for multiple comparisons using the Holm-Šídák method) or 1-way/2-way ANOVA with Tukey’s post hoc test using GraphPad Prism 8.3.0/9.2.0. Data are presented as mean ± SEM, and differences were considered significant at *P* ≤ 0.05.

### Study approval.

Animal experiments conformed to the Association for Research in Vision and Ophthalmology Statement on the Use of Animals in Ophthalmic and Vision Research and were approved by the University of Delaware Institutional Animal Care and Use Committee. Experiments using human cadaver-derived lens tissue were reviewed by the University of Delaware Institutional Review Board and were judged as exempt from review.

## Author contributions

MHS designed research studies, conducted experiments, analyzed data, and wrote the manuscript; SGN conducted experiments and analyzed data; YW designed research studies and conducted experiments; DS provided reagents and designed studies; AA provided reagents and designed studies; TDA provided reagents and designed studies; NMR conducted experiments; APF analyzed data and designed studies; and MKD designed research studies, analyzed data, and wrote the manuscript.

## Supplementary Material

Supplemental data

## Version 1 09/23/2021

In-Press Preview

## Version 2 11/08/2021

Electronic publication
