# Supplementary material for: αVβ8 integrin targeting to prevent posterior capsular opacification
Source: JCI Insight. 2021 Nov 8;6(21):e145715. doi: 10.1172/jci.insight.145715 (PMC8663568; doi:10.1172/jci.insight.145715)
Supplement: Supplemental data [file jciinsight-6-145715-s117.pdf]

Supplemental Figure 1

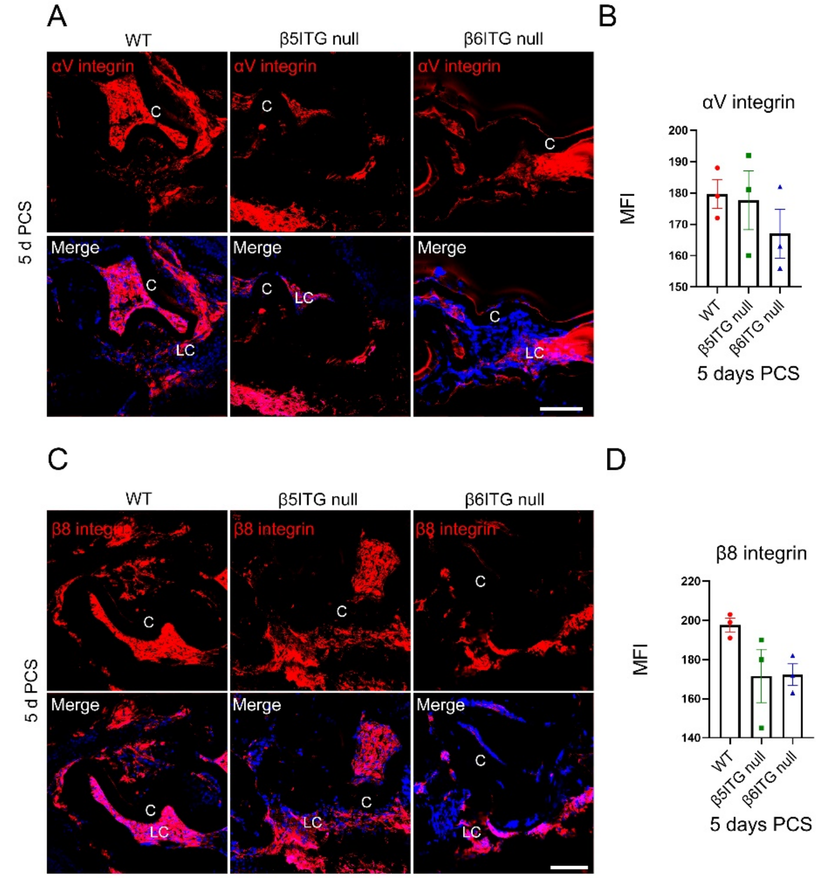

(A-D) Deletion of neither β5- nor β6 integrin affect the amount of αV- or β8- integrin protein produced by LCs PCS. All three mice strains (WT, β5ITG null, and β6ITG null) express comparable levels of αV integrin (A, B) and β8 integrin (C, D) protein at 5 d PCS (panels A & B; αV integrin, (WT vs β5ITG null, *P* = 0.981), (WT vs β6ITG null, *P* = 0.5)); (panels C & D; β8 integrin, (WT vs β5ITG null, *P* = 0.169), (WT vs β6ITG null, *P* = 0.181)). C- lens capsule, LC- lens cells, d- day, WT- wildtype, β5ITG- β5 integrin, β6ITG- β6 integrin, blue represents DNA detected by Draq5, red represents αV integrin/ β8 integrin, scale bar- 72 μm. All experiments had N = 3. Values are expressed as mean ± SEM. Asterisks (*) indicate statistically significant MFI between two groups at 5 days PCS (**P* ≤ 0.05; ***P* ≤ 0.01; ****P* ≤ 0.001); One-way ANOVA with Tukey's post hoc test. Graph legends; red (WT), green (β5ITG null), blue (β6ITG null).

Supplemental Figure 2

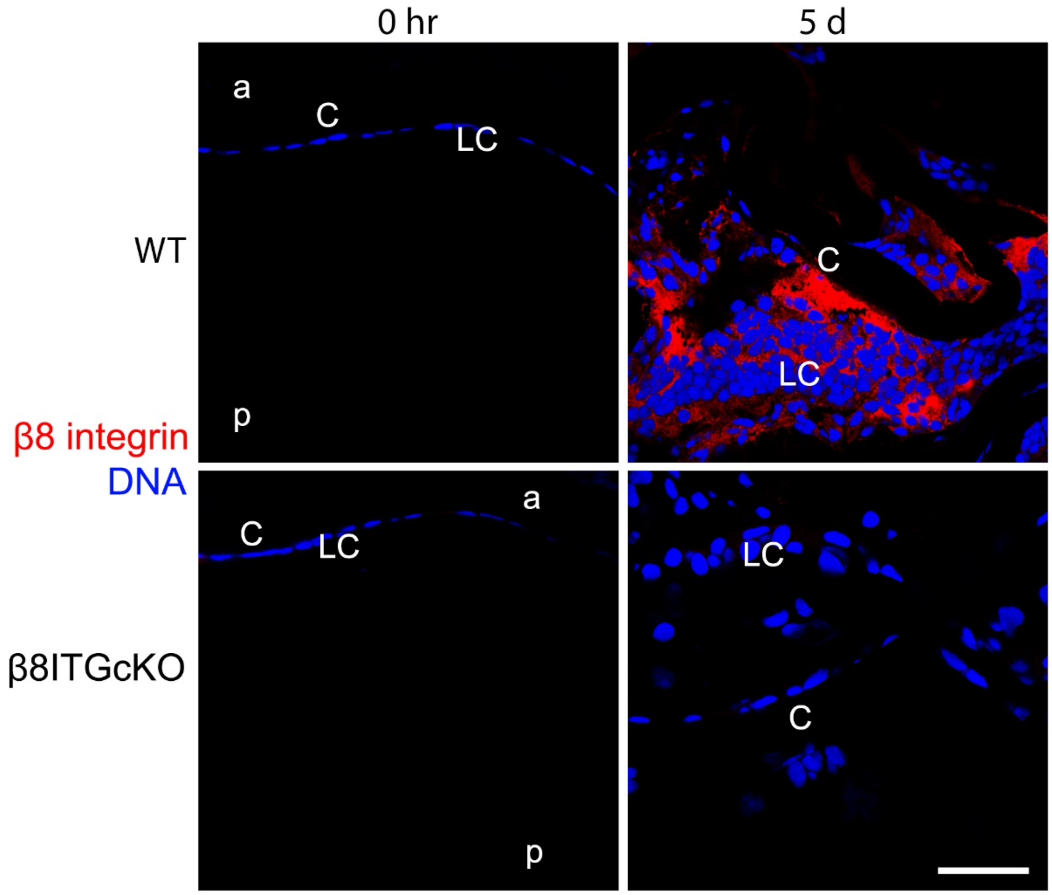

Immunofluorescence data reveals that the expression of β8 integrin protein (red) is absent both at 0 hour and 5 days post cataract surgery (PCS) in β8ITGcKO lens cells (LCs) while wildtype LCs upregulate robust levels of β8 integrin protein (Red) at 5 days PCS suggesting the successful deletion of β8 integrin gene from the lens in β8ITGcKO. C- lens capsule, LC- lens cells, a- anterior, p- posterior, d- day, hr- hour, WT- wildtype, β8ITGcKO- β8 integrin conditional knockout, blue represents DNA detected by Draq5, scale bar- 36 μm.

Supplemental Figure 3

A

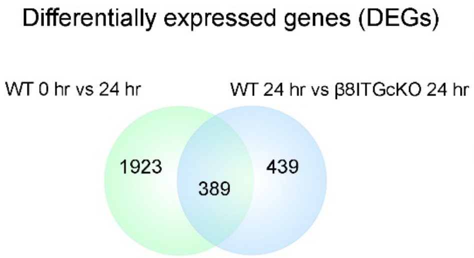

B

Differentially expressed genes between Wildtype 0 hour vs 24 hours post cataract surgery (PCS)	Total	Upregulation	Downregulation
	2312 (1923 + 389)	1273	1039

Differentially expressed genes between Wildtype 24 hours vs β8ITGcKO 24 hours post cataract surgery (PCS)	Total	Upregulation	Downregulation
	828 (439 + 389)	678	150

Biologically significant interaction (389)	Wildtype 0 hour vs 24 hours PCS (upregulation)	Wildtype 0 hour vs 24 hours PCS (downregulation)
Wildtype vs β8ITGcKO- 24 hours PCS (upregulation)	17	263
Wildtype vs β8ITGcKO- 24 hours PCS (downregulation)	102	7

(A) A Venn diagram showing that a total of 2312 genes are differentially expressed (DEGs) in wildtype (WT) remnant lens cells (LCs) at 24-hours post cataract surgery (PCS) compared to 0 hour PCS. The expression levels of 828 genes were significantly different between WT and β8ITGcKO (β8 null) LCs at 24 hours PCS. Of these, 389 DEGs which exhibit altered expression levels (either upregulated or downregulated at 24 hours PCS from 0 hour PCS) in WT LCs in response to lens fiber cell removal, show significantly altered expression (either upregulated or downregulated) in β8ITGcKO LCs at 24 hours PCS. **(B)** A breakdown of the differentially expressed genes between wildtype 0 hour and 24 hours PCS and between wildtype and β8ITGcKO LCs at 24 hours PCS is depicted in a table.

Supplemental Figure 4

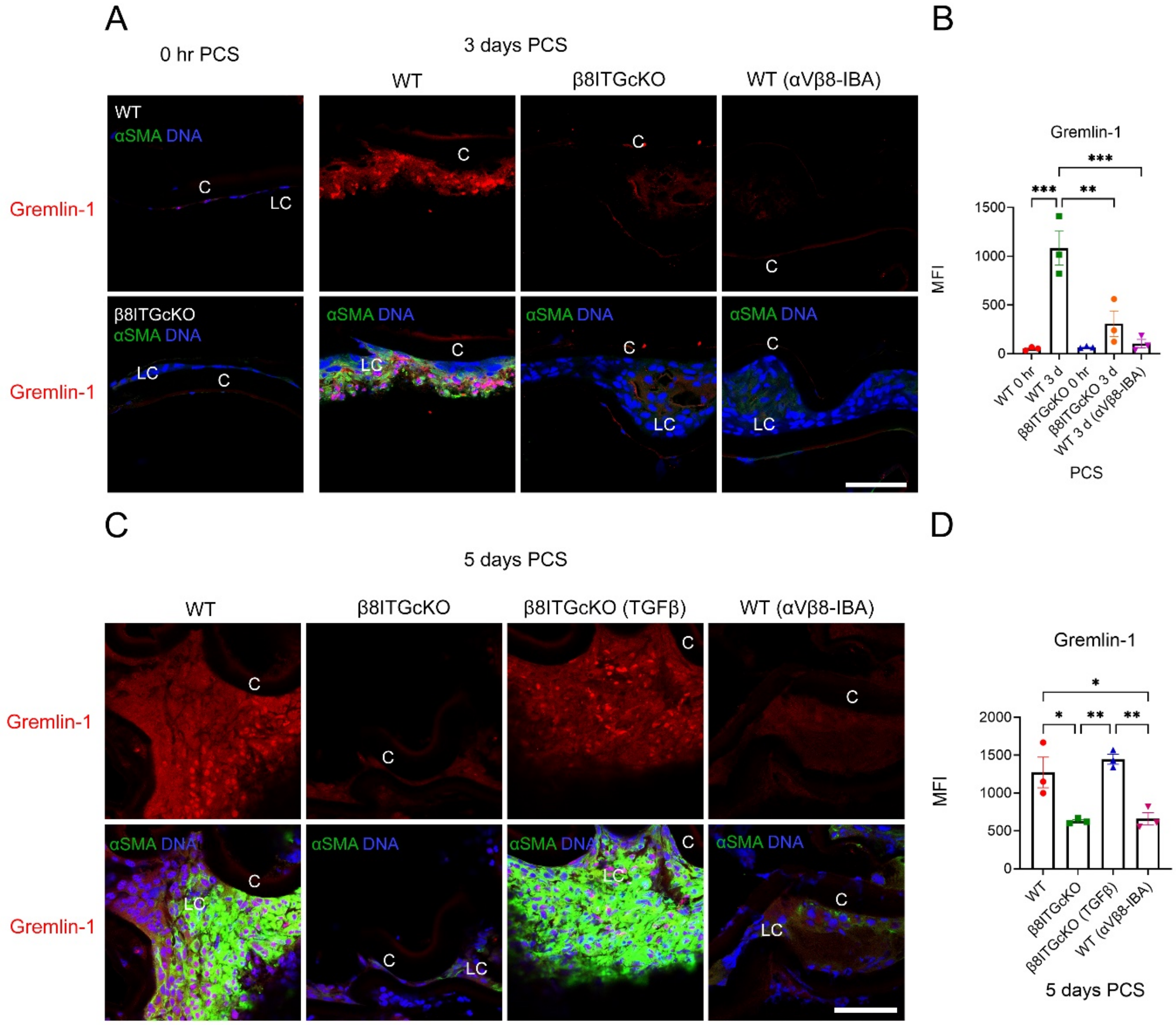

(A & B) WT LCs significantly upregulate gremlin-1 protein levels at 3 d PCS (****P* < 0.001) while β8ITGcKO LCs fails to do so compared to WT (***P* = 0.002). The addition of an αVβ8 integrin blocking antibody (αVβ8-IBA) to WT also inhibits the upregulation of gremlin-1 expression compared to WT (****P* < 0.001). **(C, D)** β8ITGcKO LCs exhibited lower gremlin-1 protein levels compared to WT at 5 days PCS (**P* = 0.017) while this phenomenon is rescued by treatment with active TGFβ1 in β8ITGcKO (TGFβ) (***P* = 0.004) LCs at 5 d PCS. Treatment of WT LCs with αVβ8- IBA resulted in a similar inhibition of gremlin-1 levels compared to WT LCs (**P* = 0.022) and β8ITGcKO LCs (*P* = 0.997).

Abbreviations; Scale bar- 35 μm, C- lens capsule, LC- remnant lens cells, MFI- mean fluorescence intensity, PCS- post cataract surgery, αVβ8-IBA- αVβ8 integrin blocking antibody, Control mice were treated with an isotype-matched antibody (anti-human αVβ3 integrin that does not cross-react with the mouse αVβ3 integrin protein); gremlin-1 (red), αSMA (green), DNA detected by Draq5 (blue). All experiments had N = 3. Values are expressed as mean $\pm$ SEM. Asterisks (*) indicate statistically significant MFI between WT and/or β8ITGcKO and/or β8ITGcKO (TGFβ) and/or WT (αVβ8-IBA) at a PCS or between two PCS time points (*$P \leq 0.05$; **$P \leq 0.01$; ***$P \leq 0.001$); Student's t-test (correct for multiple comparisons using the Holm-Šídák method) or one-way ANOVA with Tukey's post hoc test.

Graph legends; (B) red (WT 0 hour), green (WT 3 days), blue (β8ITGcKO 0 hour), orange (β8ITGcKO 3 days), purple (WT 3 days (αVβ8-IBA)); (D)- red (WT), green (β8ITGcKO), blue (β8ITGcKO (TGFβ)), purple (WT (αVβ8-IBA)).

Supplemental Figure 5

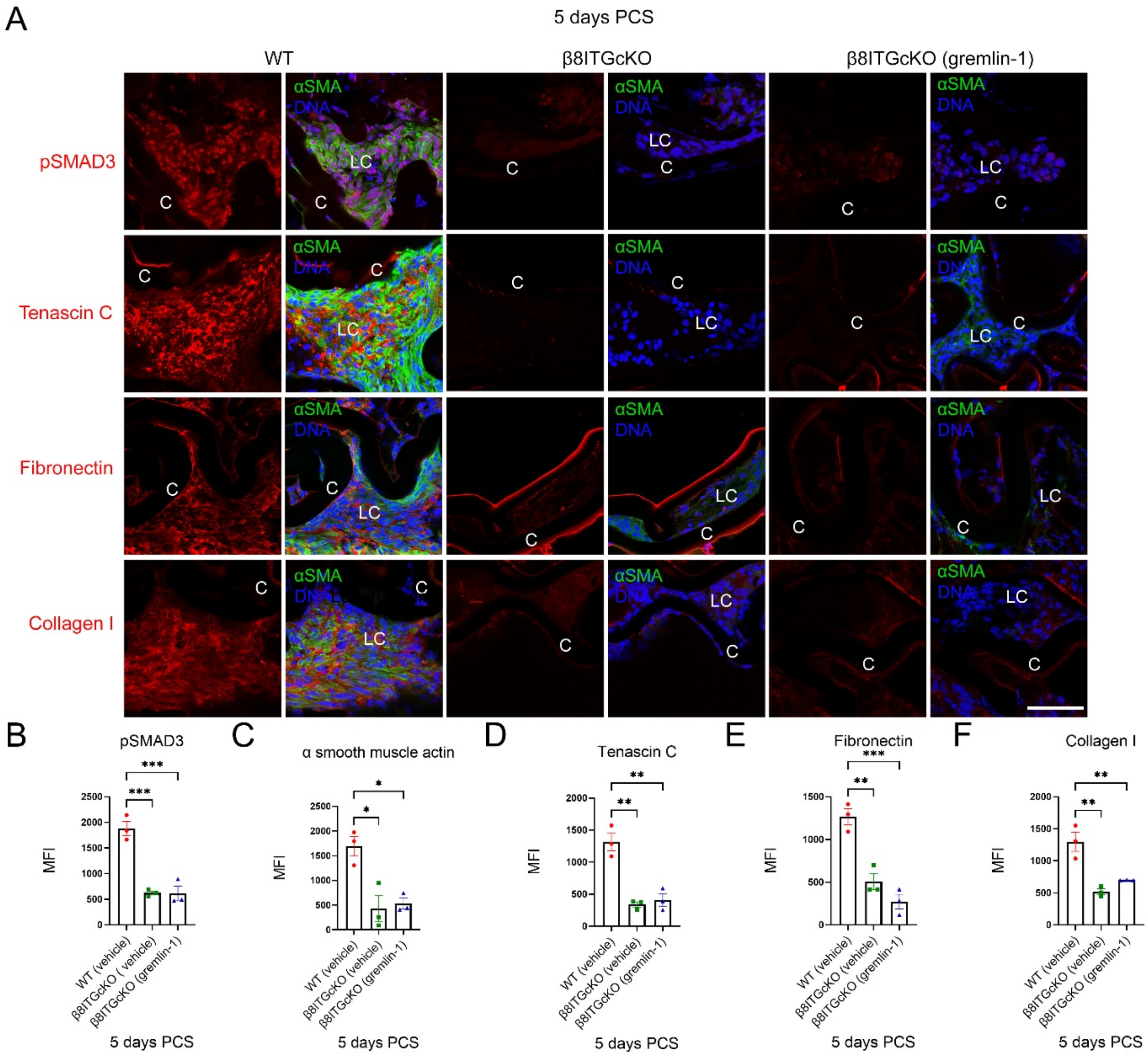

(A - F) The addition of gremlin-1 to β8ITGcKO capsular bags does not rescue the defects in LC fibrotic response measured by pSMAD3 activation (panels A & B; $P > 0.999$), αSMA (panels A & C; $P = 0.933$) , tenascin C (panels A & D; $P = 0.874$), fibronectin (panels A & E; $P = 0.241$), and collagen I (panels A & F; $P = 0.385$) at 5 d PCS compared to β8ITGcKO (vehicle).

Abbreviations: Scale bar- 35 µm, C- lens capsule, LC- remnant lens cells, MFI- mean fluorescence intensity, PCS- post cataract surgery, pSMAD3, Tenascin C, Fibronectin, Collagen I (red), αSMA (green), DNA detected by Draq5 (blue). All experiments had N = 3. Values are

expressed as mean ± SEM. Asterisks (*) indicate statistically significant MFI between WT and/or β8ITGcKO and/or β8ITGcKO (gremlin-1) at a PCS (*$P \leq 0.05$; **$P \leq 0.01$; ***$P \leq 0.001$); One-way ANOVA with Tukey's post hoc test.

Graph legends; red (WT), green (β8ITGcKO), blue (β8ITGcKO (gremlin-1)).

Supplemental Table 1: Primers and PCR conditions used to perform genotyping in this study

<table>
<tr><th>Gene</th><th>Forward Primer</th><th>Reverse Primer</th><th>PCR conditions (genotyping)</th></tr>
<tr><td>β6- integrin
(Tail) (64)</td><td>5’-TAAGTGAGTGAACTCCCTGG- 3’
(WT)

5’-CAGTAAATCGTTGTCAACAG-3’
(β6-integrin null)</td><td>5’-CAGCAATGAGTGAAAGCCA- 3’</td><td>1. Initiation/Melting- 94ºC (2 min.)
2, Denaturation- 94ºC (1 min.)
3. Annealing- 60ºC (1 min.)
4. Elongation- 72ºC (1 min.)
Steps 2-3-4 cycle in sequence-40 cycles
5. Amplification- 72ºC (5 min.)
6. Hold- 15ºC

band size –wildtype- 450 kb;
β6-integrin null- 250 kb; het- both bands.</td></tr>
<tr><td>β8- integrin
(tail) (65)</td><td>5’-GAGATGCAAGAGTGTTTACC-3’</td><td>5’-CACTTTAGTATGCTAATGATGG-3’</td><td rowspan="2">1. Initiation/Melting- 94ºC (5 min.)
2, Denaturation- 94ºC (15 sec.)
3. Annealing- 65ºC to 55 ºC (↓1ºC/cycle) (30 sec.) for first 10 cycles, next 30 cycles anneal at 55 ºC
4. Elongation- 72ºC (40 sec.)
Steps 2-3-4 cycle in sequence
5. Amplification- 72ºC (5 min.)
6. Hold- 15ºC

band size –wildtype- 250 kb;
β8-integrin floxed – 370 kb; het- both bands.

This PCR protocol is developed by MMRRC at the University of California, Davis, USA.
https://mmrrc.ucdavis.edu/protocols/014108Geno_Protocol.pdf</td></tr>
<tr><td>β8- integrin
(lens) (65)</td><td>5′-GTGGTTAAGAGCACCGATTG-3′
(F1)

5′-GAGATGCAAGAGTGTTTACC-3′
(F2)</td><td>5′-CACTTTAGTATGCTAATGATG-3′
(R1)</td></tr>
</table>

Supplemental Table 2: Primary antibodies used in this study

Primary antibody	Fixation	Blocking buffer	Primary antibody conditions
Fibronectin (ab2413, Abcam)	4% PFA	2% BSA in PBS	1:200; 1 hour at RT
Collagen I (PA5-95137, Invitrogen)	4% PFA	5% goat serum and 2% BSA in PBS	1:100; overnight at 4°C
Tenascin C (T3413, Sigma-Aldrich)	4% PFA	2% BSA in PBS	1:200; overnight at 4°C
Aquaporin0 (AB3071, Millipore)	1:1 acetone-methanol	2% BSA in PBS	1:200; overnight at 4°C,
α-SMA (1A4 F3777 & C6198, Sigma-Aldrich)	1:1 acetone-methanol	2% BSA in PBS	1:250; 1 hour at RT,
Ki 67 (D3B5, Cell Signaling)	4% PFA	Blocking buffer-5% NGS with 0.3 % TritonX-100 in PBS, Antibody buffer- 2% BSA with 0.3% TritonX-100 in PBS	1:100; overnight at 4°C
α5-integrin (ab150361, Abcam)	4% PFA	2% BSA and 5% NGS in PBS	1:200; overnight at 4°C
β1-integrin (MAB 1997, Millipore)	1:1 acetone-methanol	2% BSA in PBS	1:100; 1 hour at RT,
Phospho- S423/S425 SMAD3 (ab52903, Abcam)	4% PFA	10 min. wash in 5% BSA followed by 5% NGS, 10% horse serum and 0.3% Triton X-100 in PBS	1:100; overnight at 4°C
Gremlin-1 (PA5-13123, Invitrogen)	4% PFA	5% goat serum and 2% BSA in PBS	1:200; overnight at 4°C
αV-integrin (AB1930, Millipore Sigma)	4% PFA	5% goat serum and 2% BSA in PBS	1:200; overnight at 4°C
pFAK (44-624G, Thermofisher)	4% PFA	5% goat serum and 2% BSA in PBS	1:100; overnight at 4°C
β8 integrin (ab80673, Abcam)	4% PFA	5% goat serum and 2% BSA in PBS	1:100; overnight at 4°C
E-cadherin (24E10) Rabbit mAb #3195, Cell Signaling)	4% PFA	5% goat serum and 2% BSA in PBS	1:100; overnight at 4°C

Supplemental Table 3: Secondary antibodies and DNA dye used in this study

**Reagents**	**conditions**
Goat anti-Rabbit IgG (H+L) Cross-Adsorbed Secondary Antibody, Alexa Fluor 488 (Cat # A-11008, Invitrogen)	1:200; 1 hour at RT
Goat anti-Rat IgG (H+L) Cross-Adsorbed Secondary Antibody, Alexa Fluor 488 (Cat # A-11006, Invitrogen)	1:200; 1 hour at RT
Goat anti-Rabbit IgG (H+L) Cross-Adsorbed Secondary Antibody, Alexa Fluor 568 (Cat # A-11011, Invitrogen)	1:200; 1 hour at RT
Goat anti-Rat IgG (H+L) Cross-Adsorbed Secondary Antibody, Alexa Fluor 568 (Cat # A-11077, Invitrogen)	1:200; 1 hour at RT
Goat anti-Rabbit IgG (H+L) Cross-Adsorbed Secondary Antibody, Alexa Fluor 647 (Cat # A-21244, Invitrogen)	1:200; 1 hour at RT
Draq-5 (Biostatus Limited)	1:2000; 1 hour at RT
DAPI (Fluoropure D21490, Thermofisher)	1:2000; 1 hour at RT

Supplemental Table 4: Genes known to be involved in inflammation are upregulated by LCs by 24 hr PCS.

Gene ID	Gene description	Fold_ Change	FDR	WT_0_Hour_Avg_ FPKM	WT_24_Hour_Avg_ FPKM
S100a9	S100 calcium binding protein A9 (calgranulin B)	∞	3.92E-4	0.00	36.69
Cxcl3	chemokine (C-X-C motif) ligand 3	∞	3.92E-4	0.00	33.77
S100a8	S100 calcium binding protein A8 (calgranulin A)	∞	3.92E-4	0.00	20.44
Csf3	colony stimulating factor 3 (granulocyte)	199.24	2.76E-2	0.24	47.06
Cxcl5	chemokine (C-X-C motif) ligand 5	140.46	3.92E-4	0.31	44.06
Lcn2	lipocalin 2	123.32	3.92E-4	28.66	3533.70
Ccl6	chemokine (C-C motif) ligand 6	44.75	3.92E-4	0.57	25.32
Ptgs2	prostaglandin-endoperoxide synthase 2	29.33	3.92E-4	0.77	22.58
Ptx3	pentraxin related gene	28.99	1.05E-3	1.95	56.58
Cxcl2	chemokine (C-X-C motif) ligand 2	11.78	3.92E-4	4.36	51.38
Ier3	immediate early response 3	9.42	3.92E-4	4.76	44.90
Ctsc	cathepsin C	5.02	3.92E-4	8.48	42.60
Csf1	colony stimulating factor 1 (macrophage)	4.98	3.92E-4	6.45	32.15
Cxcl1	chemokine (C-X-C motif) ligand 1	4.96	3.92E-4	7.18	35.62
S100a6	S100 calcium binding protein A6 (calcyclin)	4.05	3.92E-4	324.59	1314.14
Ptges2	prostaglandin E synthase 2	3.77	3.92E-4	2.51	9.47

FDR- False Discovery Rate, Avg- Average, FPKM- Fragments Per Kilobase Million

∞ Fold change could not be calculated as no RNA molecules were detected in LCs at 0 hours PCS

Supplemental Table 5: Genes upregulated in LCs at 24 hr PCS that are known to be involved in fibrosis either in PCO or other systems.

Gene ID	Gene description	Fold_ Change	FDR	WT_0_Hour_Avg_ FPKM	WT_24_Hour_Avg_ FPKM
Tnc	tenascin C	175.38	3.92E-4	1.01	176.76
Grem1	gremlin 1, DAN family BMP antagonist	170.52	3.92E-4	0.97	165.86
Ecm1	extracellular matrix protein 1	79.11	3.92E-4	2.00	158.17
Tgfbi	transforming growth factor, beta induced	47.81	3.92E-4	5.80	277.16
Arg1	arginase, liver	46.89	1.93E-3	0.59	27.82
Fn1	fibronectin 1	34.21	3.92E-4	4.71	161.08
Fbln2	fibulin 2	28.91	3.92E-4	1.89	54.50
Spp1	secreted phosphoprotein 1	28.19	3.92E-4	1.02	28.89
Itga7	integrin alpha 7	23.71	3.92E-4	3.82	90.67
Tagln2	transgelin 2	10.54	3.92E-4	13.16	138.74
Nes	nestin	9.71	3.92E-4	22.12	214.74
Acta2	actin, alpha 2, smooth muscle, aorta	9.71	3.92E-4	83.37	809.26
Emp1	epithelial membrane protein 1	9.58	3.92E-4	4.66	44.63
Wisp2	WNT1 inducible signaling pathway protein 2	9.33	3.92E-4	1.32	12.35
Lox	lysyl oxidase	9.31	3.92E-4	0.57	5.32
Itga5	integrin alpha 5 (fibronectin receptor alpha)	9.06	3.92E-4	7.28	65.93
Emp3	epithelial membrane protein 3	8.01	3.92E-4	6.19	49.55
Thbs1	thrombospondin 1	6.12	3.92E-4	7.56	46.22
Runx1	runt related transcription factor 1	6.12	3.92E-4	4.21	25.72
Col1a1	collagen, type I, alpha 1	5.28	3.92E-4	2.06	10.89
E2f1	E2F transcription factor 1	3.57	3.92E-4	1.66	5.94
Ltbp1	latent transforming growth factor beta binding protein 1	3.51	3.92E-4	31.57	110.70
Tgfb1	transforming growth factor, beta 1	2.70	3.92E-4	21.88	59.05
Aebp1	AE binding protein 1	3.33	3.92E-4	32.16	107.15
Mmp14	matrix metallopeptidase 14 (membrane-inserted)	2.67	3.92E-4	7.80	20.81
Itgb1	integrin beta 1 (fibronectin receptor beta)	2.58	3.92E-4	69.11	178.41
Junb	jun B proto-oncogene	2.24	3.92E-4	35.58	79.86
Itgav	integrin alpha V	1.89	3.92E-4	49.77	93.90

FDR- False Discovery Rate, Avg- Average, FPKM- Fragments Per Kilobase Million

Supplemental Table 6: Genes that are preferentially expressed in the lens or important for the lens cells homeostasis downregulate in LCs by 24 hr PCS.

Gene ID	Gene description	Fold_ Change	FDR	WT_0_Hour_Avg_ FPKM	WT_24_Hour_Avg_ FPKM
Crygd	crystallin, gamma D	-352.63	1.15E-2	236.24	0.67
Crygb	crystallin, gamma B	-280.30	3.92E-4	460.69	1.64
Crygc	crystallin, gamma C	-75.95	3.92E-4	597.43	7.87
Lenep	lens epithelial protein	-21.99	3.92E-4	125.29	5.70
Mip	major intrinsic protein of lens fiber	-8.58	3.92E-4	440.29	51.29
Bfsp1	beaded filament structural protein 1	-8.55	3.92E-4	471.63	55.17
Lim2	lens intrinsic membrane protein 2	-7.69	3.92E-4	213.55	27.76
Fgf1	fibroblast growth factor 1	-6.76	3.92E-4	14.89	2.20
Cryba4	crystallin, beta A4	-6.70	3.92E-4	2153.80	321.65
Crybb1	crystallin, beta B1	-5.61	3.92E-4	1660.04	295.71
Crygn	crystallin, gamma N	-5.53	3.92E-4	194.88	35.23
Cryba1	crystallin, beta A1	-5.43	3.92E-4	7251.02	1335.36
Lctl	lactase-like	-4.27	3.92E-4	50.70	11.87
Gas6	growth arrest specific 6	-4.04	3.92E-4	111.42	27.56
Cryba2	crystallin, beta A2	-3.97	3.92E-4	7397.48	1862.57
Gja3	gap junction protein, alpha 3	-3.96	3.92E-4	203.81	51.49
Dkk3	dickkopf WNT signaling pathway inhibitor 3	-3.33	3.92E-4	1032.30	309.94
Cryab	crystallin, alpha B	-2.92	7.12E-3	19582.00	6705.13
Tdrd7	tudor domain containing 7	-2.84	3.92E-4	96.90	34.07
Id3	inhibitor of DNA binding 3	-2.66	3.92E-4	77.94	29.29
Foxe3	forkhead box E3	-2.34	3.92E-4	126.31	53.87
Col4a4	collagen, type IV, alpha 4	-2.31	1.15E-2	194.46	84.08
Col4a3	collagen, type IV, alpha 3	-2.29	1.93E-3	211.67	92.42
Pitx3	paired-like homeodomain transcription factor 3	-2.20	3.92E-4	56.28	25.63
Prox1	prospero homeobox 1	-2.15	3.92E-4	160.02	74.56

FDR- False Discovery Rate, Avg- Average, FPKM- Fragments Per Kilobase Million

Supplemental Table 7: Genes that normally upregulate in remnant LCs whose upregulation is attenuated in β8ITGcKO LCs at 24 hr PCS

Gene ID	WT_0 hr vs 24 hr _FC	WT_0 hr vs 24 hr_ FDR	24 hr_ WT vs β8ITGcKO_FC	24 hr_ WT vs β8ITGcKO_FDR	WT 24 hr_ Avg_ FPKM	β8ITGcKO 24 hr_ Avg _FPKM
Acod1	∞	3.92E-4	-2.42	9.36E-3	4.27	1.76
Acta2	9.71	3.92E-4	-2.18	1.01E-3	869.12	399.19
Akap2	2.27	6.93E-3	-2.41	1.01E-3	55.04	22.82
Ankrd1	14.76	3.92E-4	-4.90	1.01E-3	33.03	6.74
Anxa8	58.46	2.01E-2	-3.87	1.01E-3	22.77	5.88
Apbb1ip	11.03	3.92E-4	-2.31	1.04E-2	4.16	1.80
Apol9a	12.57	1.05E-3	-2.40	2.73E-2	4.93	2.06
Arc	2.43	3.92E-4	-2.60	1.01E-3	19.05	7.31
Asb5	4.05	1.74E-2	-5.12	2.04E-2	4.32	0.84
Blnk	6.42	3.92E-4	-2.43	1.01E-3	9.69	3.99
Calml3	2.52	3.86E-2	-15.66	6.59E-3	4.00	0.26
Car13	29.47	1.13E-2	-2.23	3.51E-2	3.80	1.71
Cbr2	5.10	3.92E-4	-2.48	4.74E-3	16.90	6.83
Cd33	22.05	3.92E-4	-3.13	1.01E-3	4.05	1.29
Cdk15	7.65	3.92E-4	-2.37	3.07E-2	4.81	2.03
Clmp	6.55	3.92E-4	-2.52	1.01E-3	6.62	2.63
Cmss1	2.72	8.97E-3	-2.02	2.62E-2	24.80	12.29
Col6a1	2.36	3.92E-4	-2.15	1.01E-3	22.96	10.67
Crabp2	19.91	3.92E-4	-3.49	1.01E-3	21.17	6.06
Csf3	199.24	2.76E-2	-4.86	1.01E-3	50.87	10.47
Cth	2.43	5.83E-3	-2.92	4.04E-3	6.35	2.17
Cxcl2	11.78	3.92E-4	-3.09	1.01E-3	55.40	17.94
Cxcl5	140.46	3.92E-4	-3.83	1.01E-3	47.41	12.39

Gene ID	WT_0 hr vs 24 hr _FC	WT_0 hr vs 24 hr_ FDR	24 hr_ WT vs β8ITGcKO_FC	24 hr_ WT vs β8ITGcKO_FDR	WT 24 hr_ Avg_ FPKM	β8ITGcKO 24 hr _Avg _FPKM
Defb1	18.52	2.71E-2	-10.79	1.68E-2	7.07	0.66
Dsg1b	2.61	3.92E-4	-5.32	1.01E-3	3.72	0.70
Dyrk3	3.41	2.47E-3	-2.09	2.23E-2	4.56	2.19
Ercc1	4.16	7.36E-4	-2.53	1.40E-2	27.49	10.88
Errfi1	2.45	3.92E-4	-2.11	1.01E-3	41.04	19.45
F3	8.71	3.92E-4	-2.24	1.01E-3	31.38	14.02
Fam25c	10.70	1.82E-2	-6.11	9.36E-3	22.71	3.71
Fgl2	10.34	3.92E-4	-3.52	1.01E-3	8.06	2.29
Gch1	2.12	9.94E-3	-2.97	1.01E-3	5.39	1.81
Grem1	170.52	3.92E-4	-2.94	1.01E-3	178.14	60.60
Gsta1	∞	3.92E-4	-3.21	4.52E-2	6.32	1.97
Gsta2	∞	3.92E-4	-3.14	1.50E-2	9.19	2.92
Hdc	14.13	1.64E-3	-4.48	5.37E-3	2.66	0.59
Hp	∞	3.92E-4	-3.27	1.01E-3	7.44	2.27
Ifit1	6.44	3.92E-4	-2.93	1.01E-3	18.61	6.35
Ifit3	5.59	3.92E-4	-3.18	1.01E-3	31.67	9.97
Ifit3b	6.57	3.92E-4	-2.60	4.74E-3	14.44	5.54
Il6ra	9.89	3.92E-4	-2.41	3.33E-3	4.18	1.73
Irak4	4.93	3.92E-4	-2.00	4.84E-2	5.46	2.72
Itga5	9.06	3.92E-4	-2.76	1.01E-3	70.95	25.72
Krt15	4.33	3.92E-4	-8.20	1.01E-3	29.50	3.60
Krt5	2.26	1.51E-2	-11.25	1.01E-3	5.66	0.50

Gene ID	WT_0 hr vs 24 hr _FC	WT_0 hr vs 24 hr_ FDR	24 hr_ WT vs β8ITGcKO_FC	24 hr_ WT vs β8ITGcKO_FDR	WT 24 hr_ Avg _FPKM	β8ITGcKO 24 hr_ Avg _FPKM
Krt6a	5.83	3.92E-4	-10.69	5.37E-3	3.60	0.34
Lbp	4.53	3.92E-4	-3.07	1.01E-3	4.58	1.49
Lgals3	3.60	3.92E-4	-4.14	1.01E-3	205.36	49.63
Lmcd1	15.38	2.04E-2	-4.13	8.35E-3	3.47	0.84
Lox	9.31	3.92E-4	-2.54	1.01E-3	5.74	2.26
Ly6a	9.13	3.92E-4	-19.01	2.62E-3	13.92	0.73
Map3k6	6.36	3.92E-4	-2.12	1.01E-3	8.41	3.97
Mmp19	4.93	9.15E-3	-2.32	4.24E-2	5.40	2.33
Mmp3	∞	3.92E-4	-2.45	1.01E-3	15.58	6.35
Mt2	2.96	3.92E-4	-2.35	1.01E-3	205.55	87.37
Nes	9.71	3.92E-4	-2.86	1.01E-3	231.45	80.99
Noct	2.71	3.92E-4	-2.16	1.01E-3	42.35	19.64
Notum	3.23	3.92E-4	-2.15	1.01E-3	10.41	4.83
Nov	4.12	3.92E-4	-3.35	1.01E-3	4.18	1.25
Nppb	30.40	4.46E-3	-2.55	2.15E-2	13.67	5.37
Oas2	12.38	3.92E-4	-2.78	5.37E-3	3.40	1.22
Ocel1	3.15	9.94E-3	-2.21	4.61E-2	4.21	1.91
Pak1	3.72	3.92E-4	-2.06	1.01E-3	10.99	5.34
Phf11d	4.79	3.92E-4	-2.80	1.01E-3	5.19	1.85
Pla2g2e	∞	3.92E-4	-10.44	4.94E-2	2.64	0.25
Prrx2	2.45	4.25E-2	-2.76	2.70E-2	4.54	1.65
Ptgs2	29.33	3.92E-4	-2.50	1.01E-3	24.39	9.75
Ptx3	28.99	1.05E-3	-3.03	1.01E-3	60.89	20.12

Gene ID	WT_0 hr vs 24 hr _FC	WT_0 hr vs 24 hr_ FDR	24 hr_ WT vs β8ITGcKO_FC	24 hr_ WT vs β8ITGcKO_FDR	WT 24 hr_ Avg _FPKM	β8ITGcKO 24 hr_ Avg_ FPKM
Pxdc1	2.93	7.36E-4	-2.02	1.96E-2	8.78	4.34
Rhox8	4.28	2.99E-3	-3.19	7.15E-3	5.99	1.88
Rnf125	20.31	3.92E-4	-2.43	1.01E-3	18.73	7.71
Rsad2	24.10	3.92E-4	-4.66	1.01E-3	8.24	1.77
S100a8	∞	3.92E-4	-3.34	1.78E-2	21.86	6.54
S100a9	∞	3.92E-4	-2.51	3.33E-3	39.28	15.67
Serpina3h	3.35	2.74E-3	-21.48	4.74E-3	4.40	0.20
Serpinb6b	3.43	3.92E-4	-4.48	1.01E-3	37.74	8.42
Serpine1	36.42	3.92E-4	-2.32	1.01E-3	432.22	186.00
Sfn	2.05	2.99E-3	-11.68	1.01E-3	14.60	1.25
Slco2a1	7.02	3.92E-4	-2.23	1.01E-3	21.12	9.47
Slfn1	79.05	2.64E-2	-2.26	7.15E-3	7.49	3.32
Slfn4	∞	3.92E-4	-3.52	1.01E-3	37.08	10.53
Slpi	∞	3.92E-4	-3.51	2.81E-2	4.82	1.37
Snai1	5.46	3.72E-3	-2.69	3.07E-2	3.46	1.29
Sprr1a	24.75	3.92E-4	-4.39	1.01E-3	82.49	18.77
Sprr2b	∞	3.92E-4	∞	1.01E-3	2.67	0.00
Stac	2.74	3.92E-4	-2.88	1.01E-3	9.28	3.22
Stat5a	2.80	3.92E-4	-2.38	1.01E-3	8.65	3.64
Syt17	2.37	2.20E-3	-3.31	1.01E-3	9.84	2.98

Gene ID	WT_0 hr vs 24 hr _FC	WT_0 hr vs 24 hr_ FDR	24 hr_ WT vs β8ITGcKO_FC	24 hr_ WT vs β8ITGcKO_FDR	WT 24 hr_ Avg _FPKM	β8ITGcKO 24 hr_ Avg_ FPKM
Tgm1	11.21	3.92E-4	-4.83	1.01E-3	12.74	2.64
Thbs1	6.12	3.92E-4	-2.23	1.01E-3	49.51	22.17
Tm4sf1	2.74	3.92E-4	-2.31	1.01E-3	106.45	46.00
Tpd52l1	2.08	5.15E-3	-2.06	5.99E-3	15.26	7.40
Trim30c	∞	3.92E-4	-5.24	3.42E-2	2.89	0.55
Tuba1c	3.97	3.92E-4	-2.27	1.01E-3	34.21	15.08
Vaultrc5	11.64	3.92E-4	-3.05	1.01E-3	123.45	40.41
Xaf1	6.28	3.92E-4	-2.23	5.37E-3	16.33	7.33

FC- Fold Change, WT- Wild Type, β8ITGcKO - β8 integrin conditional knockout, FDR- False Discovery Rate, Avg- Average, Hr- hour, FPKM- Fragments Per Kilobase Million, ∞ indicates that fold-change did not give a numerical value as FPKM of a specific gene appears 0 at WT 0 hour PCS

Supplemental Table 8: Attenuated upregulation of 23 genes known to be involved in fibrosis and inflammation was observed in remnant β8ITGcKO LCs at 24 hr PCS. Wildtype LCs typically upregulate the transcription of these genes in response to injury

Gene ID	Gene description	Fold_ Change	FDR	WT_24_Hour_ Avg_FPKM	β8ITGcKO_ 24_Hour_Avg_ FPKM
Csf3	colony stimulating factor 3 (granulocyte)	-4.86	1.01E-3	50.87	10.47
Pttg1	pituitary tumor-transforming gene 1	-4.78	1.01E-3	11.91	2.49
Mylk2	myosin, light polypeptide kinase 2, skeletal muscle8	-4.15	1.01E-3	3.49	0.84
Anxa8	annexin A8	-3.87	1.01E-3	22.77	5.88
Cxcl5	chemokine (C-X-C motif) ligand 5	-3.83	1.01E-3	47.41	12.39
S100a8	S100 calcium binding protein A8 (calgranulin A)	-3.34	1.78E-2	21.86	6.54
Ccl5	chemokine (C-C motif) ligand 5	-3.14	1.01E-3	85.11	27.08
Ptx3	pentraxin related gene	-3.03	1.01E-3	60.89	20.12
Grem1	gremlin 1, DAN family BMP antagonist	-2.94	1.01E-3	178.14	60.60
Nes	nestin	-2.86	1.01E-3	231.45	80.99
Itga5	integrin alpha 5 (fibronectin receptor alpha)	-2.76	1.01E-3	70.95	25.72
Snai1	snail family zinc finger 1	-2.69	3.07E-2	3.46	1.29
Lox	lysyl oxidaseal	-2.54	1.01E-3	5.74	2.26
S100a9	S100 calcium binding protein A9 (calgranulin B)	-2.51	3.33E-3	39.28	15.67
Ptgs2	prostaglandin-endoperoxide synthase 2	-2.50	1.01E-3	24.39	9.75
Mmp3	matrix metallopeptidase 3	-2.45	1.01E-3	15.58	6.35
Mmp19	matrix metallopeptidase 19	-2.32	4.24E-2	5.40	2.33
Thbs1	thrombospondin 1	-2.23	1.01E-3	49.51	22.17
Acta2	actin, alpha 2, smooth muscle, aorta	-2.18	1.01E-3	869.12	399.19
Tnc	tenascin C	-1.47	9.77E-2	189.85	128.98
Fn1	fibronectin 1	-1.44	1.19E-1	172.91	119.86
Itgb1	integrin beta 1 (fibronectin receptor beta)	-1.31	1.76E-1	191.15	146.02
Itgav	integrin alpha V	-1.24	3.30E-1	100.72	81.24

FDR- False Discovery Rate, Avg- Average, FPKM- Fragments Per Kilobase Million

Supplemental Table 9: Table of 60 TGFβ1 responsive genes that upregulate more robustly in injured wildtype lenses than in injured β8ITGcKO lenses. Transcription of these 60 genes is consistently upregulated in cultured primary cells stimulated with TGFβ1 (32)

Gene ID	24H_β8ITGcKO vs WT_ Fold_ Change	24H_β8ITGcKO vs WT_FDR	24H_ WT _ Avg _FPKM	24H_β8ITGcKO _Avg_ FPKM
Actn1	-1.48	4.05E-2	305.75	206.84
Adam12	-1.44	3.47E-2	27.47	19.04
Adam19	-2.59	1.84E-3	1.61	0.62
Ankrd1	-4.90	1.01E-3	33.03	6.74
Ano6	-1.65	1.01E-3	28.10	17.08
Arpc5	-1.43	3.91E-2	48.02	33.55
Cald1	-1.53	4.38E-2	365.52	238.90
Ccdc80	-1.85	1.01E-3	39.76	21.46
Cilp	-5.14	2.15E-2	2.91	0.57
Crlf1	-1.83	2.77E-2	11.14	6.10
Ctgf	-1.92	1.01E-3	269.86	140.67
Dsp	-6.95	1.01E-3	1.93	0.28
Dstn	-1.55	1.01E-3	170.58	110.38
Dynlt3	-1.59	8.35E-3	33.16	20.83
Etv6	-1.55	5.99E-3	23.02	14.88
F3	-2.24	1.01E-3	31.38	14.02
Fam114a1	-1.54	9.88E-3	30.77	19.97
Fam46a	-1.76	1.35E-2	4.07	2.31
Fermt2	-1.46	2.07E-2	87.91	60.06
Flnc	-1.72	1.01E-3	98.61	57.41

Gene ID	24H_β8ITGcKO vs WT_ Fold_ Change	24H_β8ITGcKO vs WT_FDR	24H_ WT _ Avg _FPKM	24H_β8ITGcKO_ Avg_ FPKM
Glipr2	-1.98	1.01E-3	16.96	8.58
Hras	-1.48	4.61E-2	53.01	35.93
Hspb1	-1.96	1.01E-3	63.06	32.12
Inhba	-1.69	1.84E-3	97.77	57.88
Inppl1	-1.46	2.28E-2	23.60	16.21
Itga5	-2.76	1.01E-3	70.95	25.72
Itpripl2	-1.55	4.04E-3	24.77	16.02
Ivns1abp	-1.64	9.36E-3	378.68	230.49
Krt7	-7.21	2.15E-2	2.18	0.30
Lif	-1.94	1.01E-3	33.00	17.02
Lmcd1	-4.13	8.35E-3	3.47	0.84
Lox	-2.54	1.01E-3	5.74	2.26
Msn	-1.51	3.59E-2	331.42	219.50
Myl6	-1.47	1.87E-2	451.22	306.63
Myl9	-1.77	1.01E-3	57.99	32.78
Noct	-2.16	1.01E-3	42.35	19.64
Palld	-1.63	1.01E-3	150.24	92.40
Pdlim5	-1.60	4.04E-3	34.74	21.71
Plaur	-1.47	3.59E-2	61.90	42.21
Pnp	-1.66	1.04E-2	12.19	7.33

Gene ID	24H_β8ITGcKO vs WT_ Fold_ Change	24H_β8ITGcKO vs WT_FDR	24H_ WT _ Avg _FPKM	24H_β8ITGcKO_Avg_ FPKM
Polr3d	-1.50	2.43E-2	33.30	22.24
Ppp1r14b	-1.63	5.37E-3	94.53	58.04
Prdx1	-1.53	5.37E-3	134.90	87.90
Ptgs2	-2.50	1.01E-3	24.39	9.75
Pxdc1	-2.02	1.96E-2	8.78	4.34
Rsu1	-1.46	3.59E-2	42.21	28.85
Runx1	-1.43	2.39E-2	27.58	19.29
S100a11	-1.46	2.51E-2	360.18	247.22
Serpine1	-2.32	1.01E-3	432.22	186.00
Smad7	-1.41	4.63E-2	27.48	19.51
Smim3	-1.55	2.36E-2	34.50	22.21
Snai1	-2.69	3.07E-2	3.46	1.29
Specc1	-1.54	6.59E-3	36.56	23.78
Sphk1	-1.85	1.73E-2	11.17	6.04
Tdg	-1.91	4.04E-3	9.19	4.82
Tgfb1	-1.42	3.73E-2	63.29	44.71
Tpm1	-1.76	1.01E-3	526.99	299.04
Tpm4	-1.57	5.37E-3	234.08	148.73
Trib1	-1.48	9.88E-3	51.10	34.47
Tubb6	-1.76	1.01E-3	182.83	103.71

H- Hour; FDR- False Discovery Rate; Avg- Average; FPKM- Fragments Per Kilobase Million.

Supplemental Table 10: Table of 47 TGFβ1 inhibited genes that downregulate more robustly in injured wildtype lenses than in injured β8ITGcKO lenses. Transcription of these 47 genes decreases significantly 24 hours after injury, less so in β8ITGcKO lenses than in wildtype lenses. Transcription of these 47 genes is consistently downregulated in cultured primary cells stimulated with TGFβ1(32)

Gene ID	24H_β8ITGcKO vs WT_ Fold_ Change	24H_β8ITGcKO vs WT_FDR	24H_ WT _Avg _FPKM	24H_β8ITGcKO_ Avg_ FPKM
Acss2	2.23	2.28E-2	1.42	3.18
Ank1	3.55	1.01E-3	0.57	2.01
Ankrd33b	2.67	1.01E-3	9.96	26.62
Arhgef26	4.45	1.01E-3	1.65	7.33
Bbs1	1.95	2.62E-3	2.38	4.65
Bbs2	1.88	2.00E-2	2.90	5.46
Bmper	2.23	2.23E-2	1.10	2.46
Cers4	2.03	1.01E-3	7.82	15.88
Cntnap1	2.13	1.01E-3	2.29	4.89
Dennd6b	2.47	1.01E-3	1.19	2.94
Frmpd4	1.79	1.91E-2	1.55	2.78
Fyco1	2.08	1.01E-3	14.44	30.05
Fzd5	1.54	3.07E-2	4.05	6.23
Ggt7	4.34	2.62E-3	0.66	2.85
Gpx3	1.83	5.37E-3	99.13	181.73
Gucy1b3	13.53	1.01E-3	0.24	3.31

Gene ID	24H_β8ITGcKO vs WT_ Fold_ Change	24H_β8ITGcKO vs WT_FDR	24H_ WT _Avg _FPKM	24H_β8ITGcKO_ Avg_ FPKM
Hmgb2	1.54	2.96E-2	41.03	63.09
Hsbp1l1	11.27	4.88E-2	0.13	1.49
Igfbp4	2.07	1.01E-3	15.86	32.79
Kbtbd3	2.54	1.14E-2	1.33	3.38
Kcnb1	7.22	1.01E-3	4.09	29.52
Klhl36	3.02	1.01E-3	4.24	12.81
Letm2	2.41	1.68E-2	1.32	3.18
Lpin1	1.69	3.27E-2	3.01	5.09
Magi2	1.51	4.08E-2	5.89	8.90
Map3k1	1.72	1.01E-3	17.36	29.92
Mctp1	4.13	4.74E-3	0.26	1.06
Mllt6	1.70	1.01E-3	14.48	24.56
N4bp2l1	3.51	2.85E-2	0.57	2.01
Nlgn1	2.47	1.01E-3	1.38	3.39
Osbp2	4.04	1.01E-3	5.45	22.02
Plekha6	3.59	1.01E-3	2.51	8.99
Plekhm3	1.51	3.00E-2	3.89	5.89
Ptp4a3	4.15	1.01E-3	11.49	47.66
Rufy3	1.57	2.39E-2	20.45	32.18

Gene ID	24H_β8ITGcKO vs WT_ Fold_ Change	24H_β8ITGcKO vs WT_FDR	24H_ WT _Avg _FPKM	24H_β8ITGcKO_ Avg_ FPKM
Shank2	2.14	4.74E-3	2.05	4.39
Stom	1.78	2.12E-2	4.23	7.55
Stx3	3.10	1.01E-3	11.79	36.54
Syne2	1.46	1.40E-2	14.31	20.95
Tbc1d8	4.78	1.68E-2	1.76	8.43
Tcea3	2.65	1.04E-2	3.09	8.17
Thsd7a	1.93	1.01E-3	2.26	4.35
Tnfsf10	2.08	1.68E-2	1.27	2.64
Trnp1	2.90	1.01E-3	7.16	20.77
Tub	5.13	1.01E-3	2.65	13.58
Vps13c	1.47	1.91E-2	8.72	12.85
Whamm	1.77	5.99E-3	8.41	14.91

H- Hour; FDR- False Discovery Rate; Avg- Average; FPKM- Fragments Per Kilobase Million
